# Identifying Key Predictors of Appropriate Discharge Destinations for Older Inpatients in Acute Care: Scoping Review

**DOI:** 10.2196/76582

**Published:** 2026-01-22

**Authors:** Christoph Leinert, Marina Liselotte Fotteler, Thomas Derya Kocar, Jessica Wolf, Lisa Beissel, Kathrin Grummich, Dhayana Dallmeier, Michael Denkinger

**Affiliations:** 1 Institute for Geriatric Research at Agaplesion Bethesda Ulm, Ulm University Medical Center, Zollernring 26, Ulm, 89073, Germany, 49 07311870; 2 DigiHealth Institute, Neu-Ulm University of Applied Sciences, Neu-Ulm, Germany; 3 ZB MED - Information Centre for Life Sciences, Cologne, Germany; 4 Department of Epidemiology, Boston University School of Public Health, Boston, MA, United States

**Keywords:** CDSS, clinical decision support system, continuity of care, discharge destination, discharge planning, feature selection, geriatrics, postacute care, predictor

## Abstract

**Background:**

Postacute care (PAC) services are important to ensure functional recovery and provide adequate care for geriatric inpatients in acute care. The choice between different PAC options can be challenging, and predictors for the most appropriate among diverse discharge options are warranted.

**Objective:**

We conducted a scoping review to identify predictors of appropriate discharge destinations for older adults (≥65 y) in acute care transitioning to different PAC settings and extract the most relevant predictors for different PAC settings as well as a generalizable set of predictor domains.

**Methods:**

The databases of Medline, Embase, Cochrane Central Register of Controlled Trials, PsycINFO, CINAHL, and Emcare were systematically searched for English or German literature published until February 25, 2022. A total of 3 researchers screened, extracted, and categorized the data according to domains, discharge destinations, mean age, and health care systems origin, focusing on predictors that increase the likelihood of a discharge destination (positive predictors). The Jaccard index was calculated to compare the similarity between different possible domain combinations and existing literature.

**Results:**

Of 22,382 records screened, 171 quantitative and 10 qualitative studies were included. After separating combined discharge destinations, we found 1047 predictors for different discharge destinations including nursing home (n=297, 28%), skilled nursing facility (n=223, 21%), inpatient rehabilitation (n=206, 20%), home with (n=97, 9%) or without (n=74, 7%) support, assisted living (n=63, 6%), and early inpatient rehabilitation (n=21, 2%). Of all positive predictors (n=723), age was the most frequently reported predictor (80/723, 11%). Geriatric syndromes were found more often in patients 80 years or older (121/192, 63%) and in non-US studies (174/285, 61%). The top reported predictors for discharge to nursing homes were diagnosed dementia (9/297, 3%) and deficits in instrumental activities of daily living (ADL; 10/297, 3%); for discharge to rehabilitation, the top predictors were longer length of stay (11/205, 5%) and existent cardiopulmonary disease (10/205, 5%); and for back home without support, the top predictors were good ADL (10/74, 14%) and mobility assessments (9/74, 12%). Among 20 predictor domains, 8 were most concordant with the literature: cognitive impairment, ADL, demographics, social support, hospitalization data, multimorbidity, mobility, and primary diagnosis.

**Conclusions:**

This scoping review provides a comprehensive overview of predictors for appropriate discharge decisions in older adults in acute care, stratified by destination, age, study origin, and the predictor domains most concordant with the literature. The results will be valuable to inform the choice of features for clinical decision support systems, including the training of machine learning algorithms.

## Introduction

Geriatric inpatients are at a high risk of functional decline during acute care treatment, and post-acute care (PAC) services are needed to ensure recovery [1]. Effective discharge planning has been shown to mitigate readmission rates, reduce hospital stays, lower associated costs, and enhance patient satisfaction [2]. Thus, choosing the most appropriate discharge destination is vital within geriatric comanagement, guaranteeing alignment with individualized rehabilitation and care needs. Modern health care systems present diverse PAC options, making selection challenging and highlighting the need for predictors of appropriate discharge destinations [3-6].

The World Health Organization defines continuity of care (COC) as the degree to which health care events are perceived as connected and coherent over time, aligning with patients’ health necessities and preferences [7]. COC can be further categorized into relational continuity (patient-provider relationship), informational continuity (communication), and management continuity (coordination) [8]. In the context of geriatric comanagement in acute inpatient care, the choice of the most appropriate discharge destination refers to the management continuity aspect. In addition to COC and discharge planning, various terms encompass this dimension, including integrated care, case management, or transitional care [9,10].

Existing research has focused on predictors differentiating binary outcomes [11-13]. However, there remains a need for predictors supporting decisions among multiple discharge options to not miss relevant features.

To address this gap, we conducted a scoping review to identify potential predictors for the most appropriate discharge destination for older inpatients in acute care transitioning to different PAC options such as outpatient, inpatient or early rehabilitation, skilled nursing facility (SNF), nursing home, assisted living, or home-based care with or without support. As different health care systems offer different discharge destinations and funding options, we also planned to stratify according to the different health care system origins. Our scoping review will inform the feature selection for the development of a machine learning−driven clinical decision support system (CDSS) within the “Supporting Surgery with Geriatric Co-Management and AI” (SURGE-Ahead) project by providing a broad overview of predictive measures for different discharge options [14].

## Methods

### Conceptualization

We conducted a scoping review adhering to the PRISMA-ScR (Preferred Reporting Items for Systematic Reviews and Meta-Analyses Scoping Review extension guidelines; Checklist 1) and the Joanna Briggs Institute guidance [15,16]. A review protocol was registered on Open Science Framework and was adapted after a piloting phase to focus on “predictors” instead of “predictors and outcome measures” due to a lack of specific outcome measures for COC identified [17].

### Eligibility Criteria

Using the Joanna Briggs Institute’s population-concept-context framework, our focus was on older adult inpatients 65 years and older treated in acute surgical or medical hospital departments [18]. We included only participants 65 years and older, verifying this by examining the inclusion criteria and the reported age structure of each study population. Studies were included if they reported an age range with a lower limit of 65 years or above or at least provided a mean or median of 65 years or more. Publications with unclear age demographics were conservatively excluded. We aimed to identify predictors utilized by health care professionals to determine the most appropriate PAC setting. Predictors were defined as discrete, separable parameters associated with a specific discharge destination. When faced with complex models combining multiple parameters, we included the individual parameters whenever feasible but not the complex model itself. In quantitative analyses, we further required evidence of a statistically significant effect (*P*≤.05). Studies had to involve transitions from acute care to different PAC options. German- or English-language publications were considered, excluding letters, comments, case studies, editorials, and studies primarily addressing health economic issues or involving psychiatry or rehabilitation departments for older adults. We excluded studies that chose to report composite discharge options like “discharge to nursing home or death,” as these outcomes should not be combined. Similarly, studies including palliative care services like hospice as part of a composite discharge destination were excluded, as we consider individualized decision-making essential for palliative care.

### Search Strategy

Comprehensive search strategies were developed that included different concepts for the successful choice of discharge destinations, including continuity of care, coordination of care, transition of care, integrated health care, case management, discharge planning, or rehabilitation eligibility determination. On February 25, 2022, the databases Medline (OVID interface), Embase (OVID), Cochrane Central Register of Controlled Trials (Wiley), PsycINFO (EBSCOhost), CINAHL (EBSCOhost), and Emcare (OVID) were systematically searched, guided by an experienced information specialist (KG). Search strategies for all databases searched are shown in Multimedia Appendix 1.

### Study Selection and Data Extraction

Records obtained through the database search were imported into the Covidence systematic review software (Veritas Health Innovation). Additionally, the primary publications of all reviews identified in the literature search were imported into Covidence. Duplicates were removed using the deduplication functionality of the software, followed by manual verification in unclear cases marked by the software (MLF and CL). Two out of the 4 reviewers (MLF, CL, LB, and JW) independently screened all titles and abstracts for eligibility, followed by full-text screening. Any discrepancies were resolved through adjudication by a third author. Data extraction for each article was conducted by 2 of the 3 authors independently (MLF, CL, and JW) and consolidated by the team. The extracted data encompassed study and population characteristics, health care systems origin, discharge destination, statistical analysis type, predictor, and quantitative measures of predictive strength (odds ratio, relative risk, confidence interval, *P* value) if available. The identified predictors were categorized into different domains after discussion in the review team. Data extraction for qualitative studies was conducted separately using a thematic evidence synthesis, assigning all qualitative and quantitative predictors, including representative quotes, to the predefined domains [19].

### Data Analysis

Predictive directions indicating an increased (positive) or reduced (negative) probability of discharge to a specific environment were determined from odds ratios, relative risks, or manual labeling and joint consensus when necessary (MLF and CL). The negative predictors reported in the included studies often used double negations or lacked a differentiation of discharge alternatives. To avoid misdirected results, we focused our analyses on the positive predictors.

Predictors were also stratified based on discharge destinations (outpatient, inpatient or early rehabilitation, SNF, nursing home, assisted living, or home-based care with or without support, long-term acute care setting, or other acute care setting). In studies that consolidated multiple discharge destinations into a single outcome, these were extracted separately. The direction of effect was added to the top 5 predictors per discharge destination via manual extraction from the literature and joint consensus labeling (MLF and CL). Additionally, we stratified by mean population age (<80 y or ≥80 y) and health care systems origin: predictors from Anglo-European studies with mostly publicly funded health care systems (Europe, Canada, Australia) were also analyzed separately from studies from the United States of America with a relevant proportion of pay-for-service health services [20-22].

Similar predictive factors identified across multiple studies were consolidated, whereas validated assessment tools were treated independently. In some studies, data on predictive measures were reported without using a validated assessment instrument. In these cases, we categorized the results as “no specific score.” As an example, in the activities of daily living (ADL) domain, many studies reported “decreased ADL” or similar but did not use an established assessment like the Barthel index (BI). In these cases, we categorized the reported predictors as “ADL no specific score.”

The Jaccard Index can be used to measure similarity and diversity of sample sets [23]. It was used to determine the concordance between different potential domain combinations and those reported in the reviewed studies. A higher index value signifies better agreement. Based on the 20 defined domains, we juxtaposed approximately 1 million potential combination sets (all possible combinations) to be compared with those found in the literature. The domain set with the highest average Jaccard index value was identified as a potentially generalizable combination of key predictor domains most commonly associated with each other and existing literature.

Descriptive statistics and data manipulation were carried out using Microsoft Excel 2019 (Microsoft). We used Python 3.12.2 for the analysis, using the itertools standard library for generating combinations and set class methods for calculating the Jaccard index. For loading the data from Excel, the pandas 2.2.1 library was used.

## Results

### Overview

**Figure 1.**
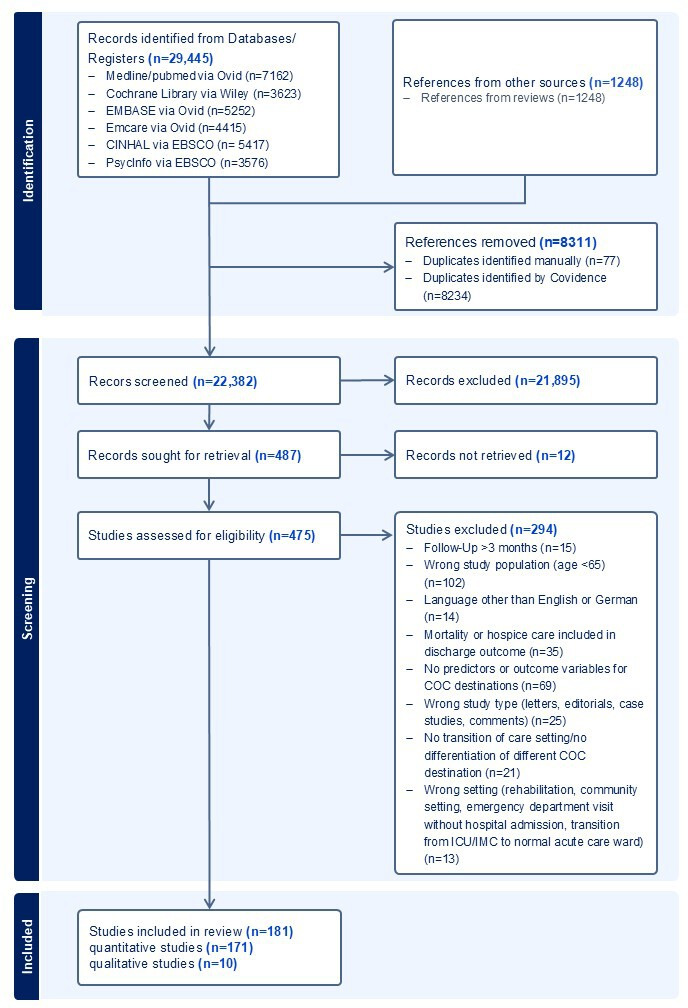
PRISMA-ScR (Preferred Reporting Items for Systematic Reviews and Meta-Analyses Extension for Scoping Reviews) flowchart. COC: continuity of care; ICU: intensive care unit; IMC: intermediate care unit.

Our search yielded 22,382 database entries after removing duplicates. After title or abstract screening, we assessed the full texts of 475 studies for eligibility and included 181 studies (n=171 quantitative studies, n=10 qualitative studies). Figure 1 shows the PRISMA flow chart of the scoping review. All included studies are listed in Multimedia Appendix 2.

The included studies were published between 1984 and 2022. Most of the 181 studies originated from North America (USA: n=84, 46%; Canada: n=14, 8%). Europe accounted for approximately one-third (n=60, 33%), including studies from the United Kingdom (n=11, 6%), the Netherlands (n=11, 6%), Switzerland (n=8, 4%), Germany (n=7, 4%), Italy (n=5, 3%), and Spain (n=3, 2%). The remaining 15% were conducted in other parts of the world, including Australia (n=14, 8%) and Japan (n=7, 4%).

A total of 275 (in part combined) settings were described, and most studies (n=161, 58.5%) were conducted in surgical settings such as trauma or orthogeriatrics (n=75, 27%) and general or visceral surgery (n=28, 10%). Other prevalent settings were geriatrics (n=29, 11%) and general or internal medicine (n=27, 10%). In 7% (n=19) of settings, no clinical department was specified. The included studies encompass data from 6,357,026 participants with a mean age of 77.42 years.

### Quantitative Studies

In the 171 quantitative studies, a total of 856 predictors for various discharge destinations were identified. More than half of these studies were prospective (88/171, 51.5%), while the remaining utilized a retrospective (78/171, 46%) or mixed approach (5/171, 3%). Most reported predictors were positive predictors (723/856, 84.5%). About two-thirds of the studies (116/171, 68%) reported the participants’ mean age, with 55 of 171 (32%) studies having a mean age of 80 years or older and 61 (36%) having a mean age younger than 80 years.

### Predictors

Age emerged as the most prevalent predictor across all categories. The 10 most frequent positive predictors are listed in Table 1 for all studies, and stratified by mean age <80 years, ≥80 years, US and Anglo-European studies.

**Table 1.** Top 10 positive predictors for continuity of care of all the included studies, stratified by mean age (<80 vs ≥80 y) and US and Anglo-European origin^a^.

Predictor	All (n=723), n (%)	<80 years mean age (n=274), n (%)	≥80 years mean age (n=192), n (%)	US (n=413), n (%)	Anglo-European (n=285), n (%)
Age	80 (11)	34 (12)	20 (10)	50 (12)	28 (10)
Wound problems	25 (4)	23 (8)	—^b^	25 (6)	—
Cardiopulmonary disease	24 (3)	16 (6)	—	23 (6)	—
ADL^c^ NSS^d^	23 (3)	10 (4)	—	19 (5)	—
Length of stay	22 (3)	6 (2)	—	—	14 (5)
Infectious disease	17 (2)	16 (6)	—	16 (4)	—
Number of comorbidities	16 (2)	11 (4)	—	15 (4)	—
ASA^e^ score [24] (multimorbidity)	16 (2)	9 (3)	—	13 (3)	—
Falls	14 (2)	—	5 (3)	9 (2)	7 (3)
Frailty index^f^	14 (2)	8 (3)	—	—	—
Female sex	14 (2)	—	6 (3)	9 (2)	—
Mobility NSS	—	7 (3)	5 (3)	9 (2)	—
Caregiver support	—	6 (2)	—	—	—
Deep vein thrombosis diagnosis	—	6 (2)	—	—	—
Source of admission	—	6 (2)	—	—	—
IADL^g^ NSS	—	—	9 (5)	—	9 (3)
Problems personal hygiene	—	—	9 (5)	—	7 (3)
Katz score [25] (ADL)	—	—	8 (4)	—	11 (4)
Charlson comorbidity index [26]	—	—	7 (4)	—	—
Dementia diagnosis	—	—	6 (3)	—	7 (3)
Cognitive deficits NSS	—	—	5 (3)	—	—
Living with or without companion	—	—	5 (3)	—	7 (3)
Barthel index [27] (ADL)	—	—	—	—	7 (3)
Injury severity score [28]	—	—	—	—	7 (3)

a n: number of extracted positive predictors, % proportion of extracted positive predictors in each group.

b Not applicable.

c ADL: activities of daily living.

d NSS: no specific score.

e ASA: American Society of Anesthesiologists.

f Frailty index: different assessments possible.

g IADL: instrumental activities of daily living.

### Discharge Destinations

We analyzed predictor-destination sets (n=1047) across 10 different discharge options. A discharge to nursing homes emerged as the most common option (n=297, 28%), followed by SNF (n=223, 21%), inpatient rehabilitation (n=206, 20%), discharge home with support (n=97, 9%) and without support (n=74, 7%), and assisted living (n=63, 6%). Less frequently found were predictors related to early inpatient geriatric rehabilitation (n=21, 2%), long-term acute care hospitals (n=23, 2%), and other acute care hospitals (n=18, 2%).

**Figure 2.**
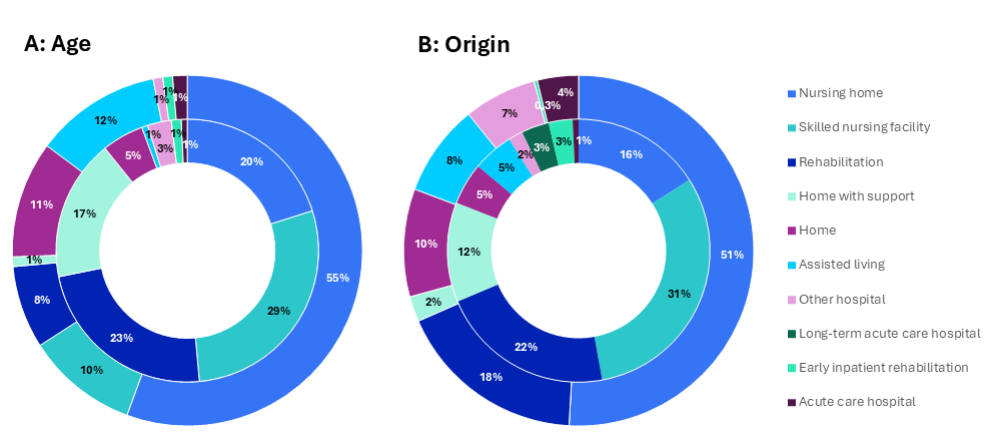
Predictors stratified by discharge destination. (A) Proportion of discharge destinations of studies stratified with mean age <80 years (inner circle, n=408) and ≥80 years (outer circle, n=223). (B) Proportion of discharge destinations of studies originating from the United States (inner circle, n=677) and Anglo-European countries (outer circle, n=342).

Nursing homes were the predominant destination in the oldest old category (≥80 y: 124/223, 55% vs <80 y: 82/408, 20%), and inpatient rehabilitation (<80 y: 95/408, 23% vs. ≥80 y: 17/223, 8%) and SNF (<80 y: 116/408, 29% vs ≥80 y: 23/223, 10%) were the main destination in the younger old category (see Figure 2A). In Anglo-European studies, the most frequently reported discharge destination was nursing home (174/342, 51%) compared to SNFs (221/677, 31%) in US studies (see Figure 2B).

### Predictors for Different Discharge Destinations

Age emerged as the predominant positive predictor for most discharge options (Table 2). Nevertheless, for a discharge home, the BI was found more often. Because of the scarcity of predictors in the categories early inpatient rehabilitation, acute care hospital, long-term acute care hospital, and other hospitals, no further stratified analysis was done for these destinations.

**Table 2.** Top 5 positive predictors for 6 discharge destinations^a^.

Predictor	Nursing home (n=297), n (%)	Skilled nursing facility (n=211), n (%)	Rehabilitation (n=205), n (%)	Home with support (n=97), n (%)	Home (n=74), n (%)	Assisted living (n=63), n (%)
Age (older)^b^	33 (11)	24 (11)	25 (12)	10 (10)	—^c^	4 (6)
IADL^d^ NSS^e^ (dependent on the instrument used)	10 (3)	—	—	—	—	—
Dementia diagnosis (yes)	9 (3)	—	—	—	—	—
Length of stay (longer)	9 (3)	—	11 (5)	4 (4)	—	—
Sex (female)	9 (3)	—	—	—	—	4 (6)
ADL^f^ NSS (dependent on the instrument used)	8 (3)	8 (4)	9 (4)	4 (4)	—	—
Cardiopulmonary disease (yes)	—	11 (5)	10 (5)	9 (9)	—	—
Wound problem (yes)	—	11 (5)	10 (5)	6 (6)	—	—
Number of comorbidities (higher)	—	8 (4)	—	4 (4)	—	—
Frailty index (frailer)	—	8 (4)	—	—	—	—
Infectious disease (yes)	—	—	—	6 (6)	—	—
Age (younger)	—	—	—	—	4 (5)	—
Barthel index [27] (higher)	—	—	—	—	6 (8)	—
Katz score [25] (ADL) (higher)	—	—	—	—	4 (5)	—
Cumulated ambulation score [29] (higher)	—	—	—	—	3 (4)	—
De Morton Mobility Index [30] (higher)	—	—	—	—	3 (4)	—
Short physical performance battery [31] (higher)	—	—	—	—	3 (4)	—
ICU^g^ treatment (yes)	—	—	—	—	—	4 (6)
Older people’s QoL^h^ questionnaire [32] (low)	—	—	—	—	—	4 (6)
Anesthesia or ICU treatment procedure (yes)	—	—	—	—	—	3 (5)

a n: number of extracted positive predictors, % proportion of extracted positive predictors in each group.

b Direction of effect in parentheses.

c Not applicable.

d IADL: instrumental activities of daily living.

e NSS: no specific score.

f ADL: activities of daily living.

g ICU: intensive care unit.

h QoL: quality of life.

Negative predictors were a minority (133/856, 15.5%) and very heterogeneous, and often seemed not useful for the identification of the most appropriate discharge destinations, such as ethnicity or male sex for discharge to rehabilitation. More relevant predictors were the unavailability of caregivers for discharge to a nursing home, a hip fracture diagnosis, or a higher frailty index, not allowing for discharge home.

A detailed overview of the extracted evidence stratified by mean age, study type, study origin, and discharge destination is shown in Multimedia Appendix 3 and, according to the domains, positive as well as negative predictors, predictive strength indicators, and corresponding literature references in Multimedia Appendix 4.

### Predictor Domains

**Figure 3.**
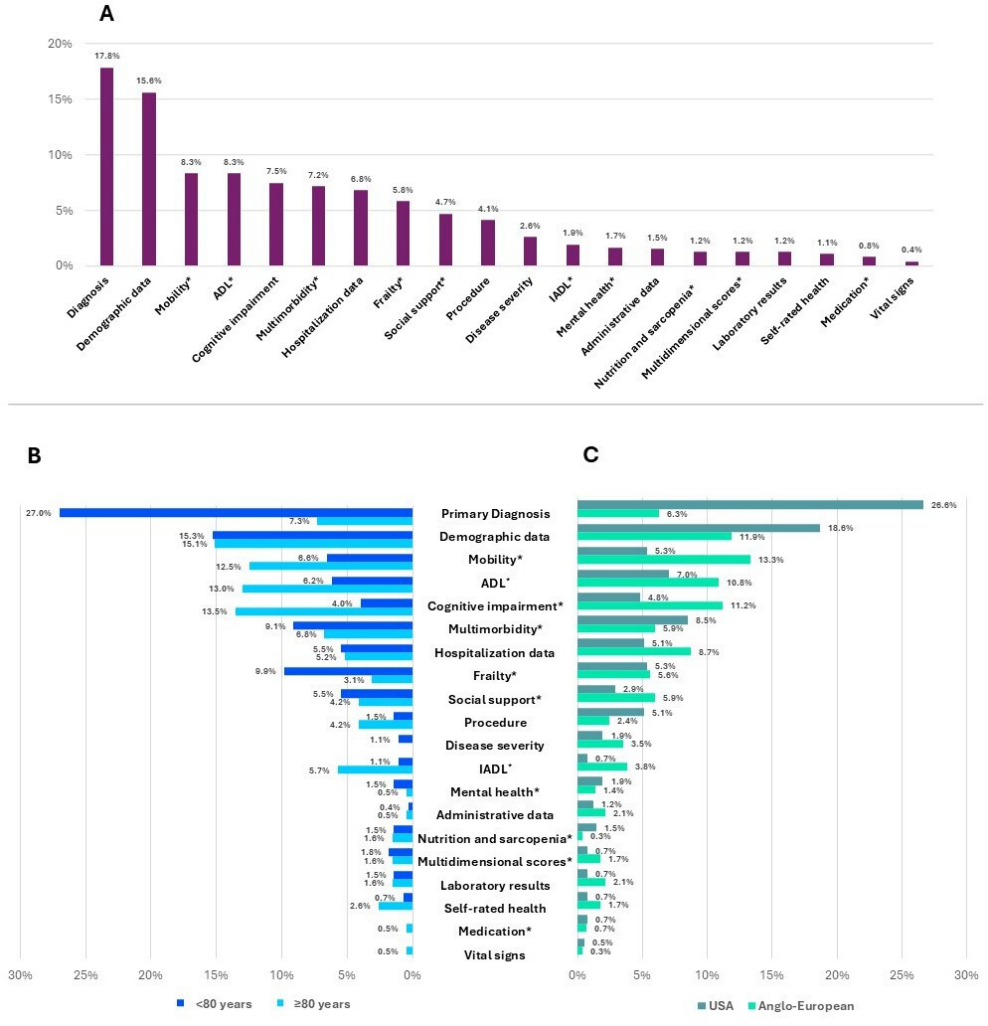
Predictor domains. (A) Dark red columns: proportion of predictors across predictor domains (n=723). (B) Proportion of predictors across all predictor domains stratified by age. Dark blue bars: mean age <80 years (n=274); light blue bars: mean age ≥80 years (n=192). (C) Proportion of predictors across all predictor domains stratified by study origin. Dark green bars: US origin (n=413); light green bars: Anglo-European origin (n=286). ADL: activities of daily living, IADL: instrumental activities of daily living. *Geriatric syndromes.

After initial data inspection, 20 predictor domains representing common health care data and geriatric syndromes were defined, and all the extracted predictors were categorized accordingly. Diagnoses (129/723, 17.8%) and demographic data (113/723, 15.6%) formed the largest proportion. The assessment domains of geriatric syndromes, such as mobility (60/723, 8%), ADL (60/723, 8%), cognitive impairment (54/723, 8%), and frailty (42/723, 6%), were commonly represented as well (Figure 3A). Geriatric syndromes were more often predictive in the oldest old category (all: 352/723, 48.7%; <80 y: 129/274, 47.1%; ≥80 y: 121/192, 63%). In contrast, the frailty domain was a more frequent predictor in the younger old category (<80 y: 27/274, 10% vs ≥80 y: 6/192, 3%), as shown in Figure 3B.

Geriatric syndromes were more often extracted from Anglo-European compared to US studies (USA: 163/413, 39.5%; Anglo-European: 174/286, 60.8%). Among these, mobility (38/286, 13%), demographic data (34/286, 12%), along with cognitive impairment (32/286, 11%), and ADL (31/286, 11%) emerged as the predominant predictor domains (Figure 3C).

In total, 93 combinations of domain sets could be identified in the literature in our review. Contrasting these sets with all potential predictor combinations of the 20 domains, the greatest concordance with the highest average Jaccard Index score of 0.28 was found for the 8 elements shown in the upper part of Table 3 as a potentially generalizable domain set. As many literature-derived sets comprise solely 1 element, based on an 8-element set, the best Jaccard Index of 2 one-element sets could be 0.125. Thus, achieving a higher index value of 0.28 with an 8-element set across all literature represents a favorable outcome.

**Table 3.** Predictor domains most concordant with the literature and potentially generalizable. Upper part: Domain set with the highest mean Jaccard index in comparison to all sets found in the literature, in alphabetical order, including the top 3 predictors of each domain. Lower part: Other domains recognized in the review, in alphabetical order, including the top 3 predictors of each domain.

Domain	Predictor examples
Domain set most concordant with the literature	
ADL^a^	ADL NSS^b^, Barthel index [27], Katz score [25]
Cognitive impairment	Dementia diagnosis, confusion assessment method [33], short portable mental status questionnaire [34]
Demographic data	Age, sex (female), ethnicity
Hospitalization data	Length of stay, ICU^c^ treatment, source of admission
Primary diagnosis	Wound problems, cardiopulmonary disease, infectious disease
Mobility	Falls, De Morton Mobility Index [30], pre-fracture mobility score [35]
Multimorbidity	American Society of Anesthesiologists score [24], number of comorbidities, Charlson comorbidity index [26]
Social support	Living alone or with companion, preadmission professional support, caregiver support
Other domains	
Administrative data	Level of care hospital, prior hospitalizations, prior ICU admission
Disease severity	Glasgow coma scale [36], injury severity score [28], injury severity NSS
IADL^d^	IADL no specific score, Lawton index [37], InterRAI Acute Care (finances) [38]
Frailty	Frailty index^e^, clinical frailty scale [39], Fried frailty scale [40]
Laboratory results	Anemia, fluid and electrolyte disorder, hypoalbuminemia
Medication	Number of medication, vitamin K antagonist therapy, therapeutic anticoagulation
Mental health	Geriatric depression scale [41], depression diagnosis, 8-item Patient Health Questionnaire [42]
Multidimensional scores	Hospital Admission Risk Profile [43], ISAR^f^ (≥2) [44] and CGA^g^ normal, multidimensional prognostic index (every 0.1 increase) [45]
Nutrition and sarcopenia	BMI [46], Mini Nutrition Assessment short form [47], sarcopenia diagnosis
Procedures	Orthopedic or trauma surgery, anesthesia or ICU treatment procedure, gastrointestinal surgery
Self-rated health	Older people’s quality of life questionnaire [32], 3-item brief health literacy screen, Short Form-12 physical component summary [48]
Vital signs	Vital capacity, respiratory rate, systolic blood pressure

a ADL: activities of daily living.

b NSS: no specific score.

c ICU: intensive care unit.

d IADL: instrumental activities of daily living.

e Frailty index: different assessments possible.

f ISAR: identification of seniors at risk.

g CGA: comprehensive geriatric assessment.

### Qualitative Studies

A total of 10 studies were found that mostly utilized semistructured interviews complemented by observational techniques and reviews of patients’ clinical records. Participants encompassed patients (n=8, 80%), health care professionals (n=8, 80%), and informal caregivers or relatives (n=2, 20%). All study characteristics can be found in Multimedia Appendix 2.

A total of 98 predictors supporting an appropriate discharge decision were extracted. Predictors spanned various discharge destinations, namely discharge in general (n=36, 37%), return to home (n=25, 26%), transfer to rehabilitation (n=15, 15%), SNF (n=14, 14%), nursing home (n=5, 5%), and home with support (n=3, 3%).

These 98 predictors were categorized into 10 domains aligning partially with those identified in the quantitative studies: patient or caregiver involvement (n=27, 28%), organizational structures (n=16, 16%), health status or morbidity (n=16, 16%), communication among health care professionals or providers (n=9, 9%), social support (n=7, 7%), staff education (n=7, 7%), regional aspects (n=6, 6%), administrative health care data (n=4, 4%), hospitalization data (n=4, 4%), and home assessment (n=2, 2%).

Within the domain of patient or caregiver involvement, effective strategies emphasize communication and respect. Notable recurring themes include discussing discharge plans with patients and caregivers in a timely manner, addressing their capability to self-manage post-discharge, providing detailed medication schedules, and honoring patient preferences. As highlighted in 1 study, “Patients reported receiving contradictory information, especially with respect to medications and how to manage their care following discharge” [49], whereas another underscored that “...elders who thought they received enough information about how to manage their care after they left the hospital reported feeling satisfied” [50].

Regarding organizational structures, clear responsibilities, including staff continuity, geriatric comanagement, and standardized discharge procedures, emerged as significant components facilitating discharge decisions. A recent study emphasized the “*...*lack of recognized decision-making tools or algorithms as a critical issue in practice” [51].

The domain health status or morbidity includes parameters akin to those observed in quantitative studies, including ADL and cognitive impairment due to dementia or delirium. In 1 study, it was cited that *“...* clinicians noted the clinical challenges of managing delirium and the need for development of patient pathways for those with delirium*”* [52].

## Discussion

### Principal Findings

This scoping review identified and synthesized predictors of appropriate PAC destinations for older adults (≥65 y), transitioning from acute care to specific discharge destinations. Based on the analysis of 181 studies (171 quantitative, 10 qualitative), key predictors varied by discharge destination. Diagnosed dementia and deficits in instrumental activities of daily living were frequently associated with discharge to nursing homes, while longer length of stay and cardiopulmonary disease predicted discharge to rehabilitation, and good performance in activities of daily living and mobility assessments favored discharge home without support. Furthermore, the review highlighted the influence of age and geographical origin, with geriatric syndromes being more prominent in those aged 80 years and older and in non-US studies. The 8 predictor domains—cognitive impairment, activities of daily living, demographics, social support, hospitalization data, multimorbidity, mobility, and primary diagnosis—demonstrated the highest concordance with existing literature. These findings provide a comprehensive overview to inform clinical decision-making and the development of clinical decision support systems, including machine learning apps, aimed at optimizing PAC planning for older adults.

### Comparison to Prior Work

Age emerged as the most frequent predictor over nearly all subgroups. Older adults ≥80 years tend to be more often discharged to nursing homes rather than rehabilitation facilities [53]. This could be due to the reduced availability and accessibility of facilities as well as misconceptions regarding their potential for improvement through these services. Investigations involving populations with a mean age of ≥80 years demonstrate an increased use of geriatric syndromes as predictors for discharge destinations, identifiable by a comprehensive geriatric assessment (CGA) [54]. Notably, frailty appears more frequently as a predictive factor in studies encompassing individuals with a mean age of <80 years. This could be because frailty was not assessed in these studies or because frailty has been mediated by age or functional parameters such as the BI [55,56]. Qualitative analysis revealed that patient or caregiver involvement was another important predictor for appropriate discharge destination, although hardly used in quantitative studies.

To address the observed heterogeneity in predictors, our review presents a higher-level categorization based on predictor domains. This provides a more standardized and manageable framework for analysis. A CGA is another established method for capturing the heterogeneity of potential limitations across geriatric domains, thereby improving the likelihood of living at home after 3 months [57]. Comparing the predictor domains most concordant with existing literature with a CGA, it stands out that 5 of the 8 predictor domains (mobility, ADL, multimorbidity, social support, and cognitive impairment) are also core elements of a CGA [54]. Therefore, a CGA supplemented by routine measurements such as current hospitalization, primary diagnosis, and demographic data forms a solid foundation for enhancing prediction accuracy of the most appropriate discharge setting and is probably one reason why it ultimately reduces discharge to higher levels of care [58].

Discharge destination choices are inherently contingent upon local health care infrastructure and accessible resources. Therefore, our analysis distinguishes between Anglo-European studies, predominantly characterized by publicly funded health care systems, and US studies with a higher proportion of pay-per-service options. Anglo-European research tends to prioritize geriatric syndromes and length of hospital stay as key predictors, whereas US studies place greater emphasis on diagnostic factors. Variations in health care systems result in disparate PAC options whose definitions diverge across settings [59,60]. For example, we found many predictors for discharge to SNF, uniquely offered in the United States, combining skilled nursing care with rehabilitative interventions, even though similar initiatives are emerging in European countries such as Germany [3,61]. Despite regional differences, parallel options exist for clinically stable yet functionally declined older patients requiring both nursing care and rehabilitation within other health care frameworks, illustrated by examples like the Australian respite residential aged care or early inpatient geriatric rehabilitation in Germany [6,62]. Regrettably, we did not find any comparative analyses examining various PAC alternatives across countries.

A lack of standardized decision support tools assisting health care professionals when deciding on the best discharge option is one of the issues that were raised in the qualitative studies [51,63,64]. This was also supported by a recent scoping review by Singh et al [65] focusing on digital health solutions facilitating transitions in care. In a systematic review on 35 CDSSs for PAC referral that included mainly studies of non-geriatric adults, Kennedy et al [13] revealed that merely 14% have been integrated into regular clinical practice, potentially hindered by constraints in time resources. Conversely, positive outcomes have emerged from specific implementations, such as a pre- or post-implementation study of a CDSS with a 2-step approach that showed a significant reduction of readmission rates [66]. Also, other binomial prediction models for discharge destinations, for example, for routine versus nonroutine discharge as in Karhade et al [67], showed promising results with an area under the curve of 0.823 in a machine learning model. However, when comparing these findings to one of the few CDSS developed for multinomial differentiation across 6 discharge destinations involving over 14,000 participants, it achieved a lower overall area under the curve of 0.685 [68]. The findings presented in this review will help to develop future multinomial COC or discharge prediction models by identifying eligible features.

### Strengths and Limitations

A key strength of our scoping review lies in the detailed differentiation of discrete prediction parameters, which enabled us to identify and statistically analyze a robust set of core predictor domains. However, our review is not without limitations, which can be categorized as pertaining to the included studies themselves and our review methodology.

We acknowledge the following limitations. The first limitation was the predominance of studies conducted in the US health care system with its specific PAC options and at least partially unmet health care needs due to some patients’ lack of insurance coverage [69,70]. To address this limitation, we compared US studies with studies from Europe, Canada, and Australia with more similarly conceptualized health care systems, even though we are aware that this simplifies the complex differences between countries in the Anglo-European group that also exist.

The second limitation was the frequent use of nonstandardized assessments that we addressed by summarizing these measures into a “no specific score” predictor. For example, the predictors “problems with personal hygiene” or “ADL no specific score” are included in both the Katz Score and BI that again report comparable information. This indicates a need for a wider use of validated assessment instruments to capture the functional status in older patients in a uniform way. When selecting assessments for the future development of a CDSS tool or compiling assessments for a CGA, these validated and established assessments should be used.

Third, many of the included studies presented composite end points of different discharge destinations with unknown proportions among the different destinations as well as different populations concerning investigated diseases and clinical settings, which limits the validity of the extracted predictors for generalizable discharge destination options. Wherever possible, we tried to extract the singular discharge destination.

Fourth, focusing on the older geriatric population might have led to an omission of other studies mainly including nongeriatric adults that might also have included valuable information on the topic. For example, a review by Kennedy et al [13] found 33 studies presenting CDSS for optimizing discharge destinations, but only 6 studies were conducted in older age populations.

Fifth, our focus on positive predictors to avoid often confusing directions of effect among the negative predictors may have resulted in an undercapture of relevant predictors. With the negative predictors representing only 15.5% of the predictors, we consider the bias of this approach to be low.

Sixth, there is a lack of further details on the extracted predictors, for example, concerning the timepoint of the assessment or the direction of effect of the predictor. These parameters have been found relevant in other studies [66]. We partially addressed this lack of detail by manually extracting the direction of effect of the most frequent predictors stratified according to different discharge destinations. The aim of a scoping review is to cover a broader topic. This approach, however, results in a large heterogeneity among the included studies and a lack of details. Thus, the analysis provided here remains on a descriptive level and offers the opportunity for future research.

Seventh, only 10 studies were included in the qualitative analysis, with most studies from the 1990s and early 2000s, limiting the informative value of the qualitative evidence synthesis.

Eighth, the broad scope of studies screened and the comprehensive analysis undertaken resulted in a literature search limited to publications up to 2022. Nevertheless, we are confident that our review of this relevant, yet relatively stable, topic remains current.

### Conclusion and Implications

This scoping review synthesized evidence on predictors of appropriate discharge destinations for older adults, highlighting the heterogeneity of factors influencing care transitions. The identified 8-domain set—including demographic data, activities of daily living, social support, hospitalization data, primary diagnosis, cognitive impairment, mobility, and multimorbidity—underscores the value of comprehensive geriatric assessment in guiding discharge planning. This work will directly inform the feature selection process for a machine learning algorithm within the SURGE-Ahead project, designed to improve discharge recommendations. Recognizing the limitations of existing data and the need for system-specific adaptation, we advocate for continued research and the implementation of evidence-based discharge planning strategies.

## Supplementary material

10.2196/76582 Multimedia Appendix 1 Search strings.

10.2196/76582 Multimedia Appendix 2 Overview of included studies and reference list. (A) Overview of included quantitative studies ordered by year of publication. P: prospective observational study (eg, cohort study); R: retrospective observational study (eg, population-based study); ES: expert survey. (B) Overview of included qualitative studies ordered by year of publication.

10.2196/76582 Multimedia Appendix 3 Predictors positive and negative stratified according to age, origin, study design, and discharge destinations.

10.2196/76582 Multimedia Appendix 4 Predictors positive and negative, domain, discharge destinations, predictive strength indicators, and corresponding literature references.

10.2196/76582 Checklist 1 PRISMA-ScR checklist.

## Abbreviations

ADL: activities of daily living
BI: Barthel index
CDSS: clinical decision support systems
CGA: Comprehensive geriatric assessment
COC: continuity of care
IADL: instrumental activities of daily living
PAC: postacute care
SNF: skilled nursing facility
SURGE-Ahead: Supporting Surgery with Geriatric Co-Management and AI
