# Supplementary material for: Identifying Key Predictors of Appropriate Discharge Destinations for Older Inpatients in Acute Care: Scoping Review
Source: Interact J Med Res. 2026 Jan 22;15:e76582. doi: 10.2196/76582 (PMC12826578; doi:10.2196/76582)
Supplement: Multimedia Appendix 1 [file ijmr-v15-e76582-s001.pdf]

Ovid Medline(r) - ALL <1946 to February 24, 2022>

Ovid MEDLINE(R) ALL <1946 to February 24, 2022>	Search
1	exp Aged/
2	((old* adj3 (person$ or people or subject$ or patient* or participant$ or adult$)) or (old* adj2 age*) or senior$).ti,ab,kf.
3	1 or 2
4	Osteoporotic Fractures/ or exp Hip Fractures/
5	(Trochanteric Fracture$ or Subtrochanteric Fracture$ or Intertrochanteric Fracture$ or hip fracture$ or femoral neck fracture$ or osteoporotic fracture$ or orthogeriatric* or ortho-geriatric* or (surgical* adj3 old*)).ti,ab,kf.
6	Geriatrics/
7	(geriatric rehabilitation or (rehabilitation* adj3 (old* or age*))).ti,ab,kf.
8	(geriatric* or gerontolog*).ti,kf. or (geriatric* or gerontolog*).ab. /freq=2
9	Geriatric Assessment/
10	Nursing Assessment/
11	(geriatric assessment$ or nursing assessment$).ti,ab,kf.
12	4 or 5 or 6 or 7 or 8 or 9 or 10 or 11
13	Forecasting/
14	(Predictor* or (prediction adj2 model$) or decision making or decision support).ti,kf. or (Predictor* or (prediction adj2 model$) or decision making or decision support).ab. /freq=3
15	"Continuity of Patient Care"/ or Patient Discharge/ or Patient Handoff/ or Patient Transfer/ or Transitional Care/
16	(care continuity or care continuum or continuity of care or continuity of patient care or continuum of care or patient care continuity or (discharge adj2 plan*) or (patient$ adj2 discharge) or (patient$ adj2 transfer) or transition care* or transitional care* or coordination of care or co-ordination of care or care coordination or care co-ordination).ti,ab,kf.
17	Case Management/
18	(case management or care pathway or integrated care pathway or care plan or care planning).ti,ab,kf.
19	"Delivery of Health Care, Integrated"/
20	((delivery adj2 integrated health care) or integrated delivery system* or integrated health care system*).ti,ab,kf.
21	Eligibility Determination/
22	((eligibility adj3 old*) or (eligibility adj3 age*) or (determin* adj3 eligibility) or (readiness adj3 old*) or (readiness adj3 age*)).ti,ab,kf.
23	(rehab* admission$ or rehab* eligibilit* or rehab* potential* or rehab* assessment$).ti,ab,kf.
24	13 or 14 or 15 or 16 or 17 or 18 or 19 or 20 or 21 or 22 or 23
25	3 and 12 and 24
26	(letter or comment or editorial).pt.
27	25 not 26

Ovid Embase

Embase <1974 to 2022 February 24>	Search
1	exp Aged/
2	((old* adj3 (person$ or people subject$ or patient* or participant$ or adult$)) or (old* adj2 age*) or senior$).tw,kf.
3	1 or 2
4	Osteoporotic Fractures/ or exp Hip Fractures/
5	(Trochanteric Fracture$ or Subtrochanteric Fracture$ or Intertrochanteric Fracture$ or hip fracture$ or femoral neck fracture$ or osteoporotic fracture$ or orthogeriatric* or ortho-geriatric* or (surgical* adj3 old*)).tw,kf.
6	Geriatrics/
7	(geriatric rehabilitation or (rehabilitation* adj3 (old* or age*))).tw,kf.
8	..nlpx "query=(geriatric* or gerontolog*).ti,kf. or (geriatric* or gerontolog*).ab. /freq=2","desiredResults=10000","minHitsDivisor=7","permitHyponyms=NO","lowestVocabularySearchLevel=none","phrasesBroken=NO","speedWanted=NoHypos","comment=Including Related Terms","elimEnable=NO","constraintMinTerms=2"
9	Geriatric Assessment/
10	Nursing Assessment/
11	(geriatric assessment$ or nursing assessment$).tw,kf.
12	4 or 5 or 6 or 7 or 8 or 9 or 10 or 11
13	Forecasting/
14	..nlpx "query=(Predictor* or (prediction adj2 model$) or decision making or decision support).ti,kf. /freq=3 or (Predictor* or (prediction adj2 model$) or decision making or decision support).ab. /freq=3","desiredResults=10000","minHitsDivisor=7","permitHyponyms=NO","lowestVocabularySearchLevel=none","phrasesBroken=NO","speedWanted=NoHypos","comment=Including Related Terms","elimEnable=NO","constraintMinTerms=2"
15	"Continuity of Patient Care"/ or Patient Discharge/ or Patient Handoff/ or Patient Transfer/ or Transitional Care/
16	(care continuity or care continuum or continuity of care or continuity of patient care or continuum of care or patient care continuity or (discharge adj2 plan*) or (patient$ adj2 discharge) or (patient$ adj2 transfer) or transition care* or transitional care* or coordination of care or co-ordination of care or care coordination or care co-ordination).tw,kf.
17	Case Management/
18	(case management or care pathway or integrated care pathway or care plan or care planning).tw,kf.
19	"Delivery of Health Care, Integrated"/
20	((delivery adj2 integrated health care) or integrated delivery system* or integrated health care system*).tw,kf.
21	Eligibility Determination/
22	((eligibility adj3 old*) or (eligibility adj3 age*) or (determin* adj3 eligibility) or (readiness adj3 old*) or (readiness adj3 age*)).tw,kf.
23	(rehab* admission$ or rehab* eligibilit* or rehab* potential* or rehab* assessment$).tw,kf.
24	13 or 14 or 15 or 16 or 17 or 18 or 19 or 20 or 21 or 22 or 23
25	3 and 12 and 24
26	(letter or comment or editorial).pt.
27	25 not 26
28	limit 27 to embase

Cochrane Central Register of Controlled Trials (Wiley)

Cochrane Library Date Run: 24/02/2022	Search

#1	MeSH descriptor: [Aged] explode all trees
#2	((old* NEAR/3 (person$ or people or subject$ or patient* or participant$ or adult$)) or (old* adj2 age*) or senior?):ti,ab,kw
#3	#1 OR #2
#4	[mh "Osteoporotic Fractures"] OR [mh "Hip Fractures"] explode all trees
#5	(Trochanteric Fracture$ or Subtrochanteric Fracture$ or Intertrochanteric Fracture$ or hip fracture$ or femoral neck fracture$ or osteoporotic fracture$ or orthogeriatric* or ortho-geriatric* or (surgical* NEAR/3 old*)):ti,ab,kw
#6	[mh Geriatrics]
#7	(geriatric rehabilitation or (rehabilitation* NEAR/3 (old* or age*))):ti,ab,kw
#8	geriatric* OR gerontolog*:ti,kw
#9	[mh "Geriatric Assessment"]
#10	[mh "Nursing Assessment"]
#11	(geriatric assessment$ or nursing assessment$):ti,ab,kw
#12	#4 OR #5 OR #6 OR #7 OR #8 OR #9 OR #10 OR #11
#13	[mh ^Forecasting]
#14	(Predictor* or (prediction NEAR/2 model$) or decision making or decision support):ti,kw
#15	[mh "Continuity of Patient Care"] OR [mh "Patient Discharge"] OR [mh "Patient Handoff"] OR [mh "Patient Transfer"] OR [mh "Transitional Care"]
#16	(care continuity or care continuum or continuity of care or continuity of patient care or continuum of care or patient care continuity or (discharge NEAR/2 plan*) or (patient$ NEAR/2 discharge) or (patient$ NEAR/2 transfer) or transition care* or transitional care* or coordination of care or co-ordination of care or care coordination or care co-ordination):ti,ab,kw
#17	[mh "Case Management"]
#18	(case management or care pathway or integrated care pathway or care plan or care planning):ti,ab,kw
#19	[mh "Delivery of Health Care, Integrated"]
#20	((delivery NEAR/2 integrated health care) or integrated delivery system* or integrated health care system*):ti,ab,kw
#21	[mh "Eligibility Determination"]
#22	((eligibility NEAR/3 old*) or (eligibility NEAR/3 age*) or (determin* NEAR/3 eligibility) or (readiness NEAR/3 old*) or (readiness NEAR/3 age*)):ti,ab,kw
#23	(rehab* admission$ or rehab* eligibilit* or rehab* potential* or rehab* assessment$):ti,ab,kw
#24	#13 OR #14 OR #15 OR #16 OR #17 OR #18 OR #19 OR #20 OR #21 OR #22 OR #23
#25	#3 AND #12 AND #24
#26	(letter:pt OR comment:pt OR editorial:pt)
#27	#25 NOT #26

PsychInfo (EBSCOhost)

# Search Date: 24-02-2022	Query - Publication Type: All Journals
S19	S17 NOT S18
S18	(letter OR comment OR editorial)
S17	S7 AND S16
S16	S8 OR S9 OR S10 OR S11 OR S12 OR S13 OR S14 OR S15

S15	TI ((rehab* admission? OR rehab* eligibilit* OR rehab* potential* OR rehab* assessment?)) OR AB ((rehab* admission? OR rehab* eligibilit* OR rehab* potential* OR rehab* assessment?))
S14	TI (((eligibility N3 old*) OR (eligibility N3 age*) OR (determin* N3 eligibility) OR (readiness N3 old*) OR (readiness N3 age*))) OR AB (((eligibility N3 old*) OR (eligibility N3 age*) OR (determin* N3 eligibility) OR (readiness N3 old*) OR (readiness N3 age*)))
S13	TI (((delivery N2 integrated health care) OR integrated delivery system* OR integrated health care system*)) OR AB (((delivery N2 integrated health care) OR integrated delivery system* OR integrated health care system*))
S12	TI ((“case management” OR “care pathway” OR “integrated care pathway” OR “care plan” OR “care planning”)) OR AB ((“case management” OR “care pathway” OR “integrated care pathway” OR “care plan” OR “care planning”))
S11	DE "Case Management"
S10	TI ((care continuity OR care continuum OR continuity of care OR continuity of patient care OR continuum of care OR patient care continuity OR (discharge N2 plan*) OR (patient? N2 discharge) OR (patient? N2 transfer) OR transition care* OR transitional care* OR coORdination of care OR co-ordination of care OR care coordination OR care co-ordination)) OR AB ((care continuity OR care continuum OR continuity of care OR continuity of patient care OR continuum of care OR patient care continuity OR (discharge N2 plan*) OR (patient? N2 discharge) OR (patient? N2 transfer) OR transition care* OR transitional care* OR coORdination of care OR co-ordination of care OR care coordination OR care co-ordination))
S9	TI ("Continuity of Patient Care" OR “Patient Discharge” OR “Patient Handoff” OR “Patient Transfer” OR “Transitional Care”) OR AB ("Continuity of Patient Care" OR “Patient Discharge” OR “Patient Handoff” OR “Patient Transfer” OR “Transitional Care”)
S8	TI ((Predictor* or (prediction N2 model?) or decision making or decision support)) OR KW ((Predictor* or (prediction N2 model?) or decision making or decision support))
S7	(S1 OR S2 OR S3 OR S4 OR S5 OR S6)
S6	TI ((geriatric assessment? or nursing assessment?)) OR AB ((geriatric assessment? or nursing assessment?))
S5	DE "Geriatric Assessment"
S4	TI ((geriatric* or gerontolog*)) OR KW ((geriatric* or gerontolog*))
S3	TI ((geriatric rehabilitation or (rehabilitation* N3 (old* or age*)))) OR ((geriatric rehabilitation or (rehabilitation* N3 (old* or age*))))
S2	DE "Geriatrics"
S1	TI ((Trochanteric Fracture? or Subtrochanteric Fracture? or Intertrochanteric Fracture? or hip fracture? or femoral neck fracture? or osteoporotic fracture? or orthogeriatric* or ortho-geriatric* or (surgical* N3 old*))) OR AB ((Trochanteric Fracture? or Subtrochanteric Fracture? or Intertrochanteric Fracture? or hip fracture? or femoral neck fracture? or osteoporotic fracture? or orthogeriatric* or ortho-geriatric* or (surgical* N3 old*)))

CINAHL (EBSCOhost)

#	Query	Limiters/Ex-panders	**Last Run on** February 22, 2022

S31	S3 AND S14 AND S30	Expanders - Apply equivalent subjects<br>Search modes - Find all my search terms	Interface - EBSCOhost Research Databases<br>Search Screen - Advanced Search<br>Database - CINAHL
S30	S15 OR S16 OR S17 OR S18 OR S19 OR S20 OR S21 OR S22 OR S23 OR S24 OR S25 OR S26 OR S27 OR S28 OR S29	Expanders - Apply equivalent subjects<br>Search modes - Find all my search terms	Interface - EBSCOhost Research Databases<br>Search Screen - Advanced Search<br>Database - CINAHL
S29	((TI "rehab* admission?" OR AB "rehab* admission?" OR SU "rehab* admission?") OR (TI "rehab* eligibilit*" OR AB "rehab* eligibilit*" OR SU "rehab* eligibilit*") OR (TI "rehab* potential*" OR AB "rehab* potential*" OR SU "rehab* potential*") OR (TI "rehab* assessment?" OR AB "rehab* assessment?" OR SU "rehab* assessment?"))	Expanders - Apply equivalent subjects<br>Search modes - Find all my search terms	Interface - EBSCOhost Research Databases<br>Search Screen - Advanced Search<br>Database - CINAHL
S28	(((TI eligibility OR AB eligibility OR SU eligibility) N3 (TI old* OR AB old* OR SU old*)) OR ((TI eligibility OR AB eligibility OR SU eligibility) N3 (TI age* OR AB age* OR SU age*)) OR ((TI determin* OR AB determin* OR SU determin*) N3 (TI eligibility OR AB eligibility OR SU eligibility)) OR ((TI readiness OR AB readiness OR SU readiness) N3 (TI old* OR AB old* OR SU old*)) OR ((TI readiness OR AB readiness OR SU readiness) N3 (TI age* OR AB age* OR SU age*)))	Expanders - Apply equivalent subjects<br>Search modes - Find all my search terms	Interface - EBSCOhost Research Databases<br>Search Screen - Advanced Search<br>Database - CINAHL
S27	(MH "Eligibility Determination")	Expanders - Apply equivalent subjects<br>Search modes - Find all my search terms	Interface - EBSCOhost Research Databases<br>Search Screen - Advanced Search<br>Database - CINAHL
S26	(((TI delivery OR AB delivery OR SU delivery) N2 (TI "integrated health care" OR AB "integrated health care" OR SU "integrated health care")) OR (TI "integrated delivery system*" OR AB "integrated delivery system*" OR SU "integrated delivery system*") OR (TI "integrated health care system*" OR AB "integrated health care system*" OR SU "integrated health care system*"))	Expanders - Apply equivalent subjects<br>Search modes - Find all my search terms	Interface - EBSCOhost Research Databases<br>Search Screen - Advanced Search<br>Database - CINAHL
S25	(MH "Delivery of Health Care, Integrated")	Expanders - Apply equivalent subjects<br>Search modes - SmartText Searching	Interface - EBSCOhost Research Databases<br>Search Screen - Advanced Search<br>Database - CINAHL

S24	((TI "case management" OR AB "case management" OR SU "case management") OR (TI "care pathway" OR AB "care pathway" OR SU "care pathway") OR (TI "integrated care pathway" OR AB "integrated care pathway" OR SU "integrated care pathway") OR (TI "care plan" OR AB "care plan" OR SU "care plan") OR (TI "care planning" OR AB "care planning" OR SU "care planning"))	Expanders - Apply equivalent subjects<br>Search modes - Find all my search terms	Interface - EBSCOhost Research Databases<br>Search Screen - Advanced Search<br>Database - CINAHL
S23	(MH "Case Management")	Expanders - Apply equivalent subjects<br>Search modes - Find all my search terms	Interface - EBSCOhost Research Databases<br>Search Screen - Advanced Search<br>Database - CINAHL
S22	((TI "care continuity" OR AB "care continuity" OR SU "care continuity") OR (TI "care continuum" OR AB "care continuum" OR SU "care continuum") OR (TI "continuity of care" OR AB "continuity of care" OR SU "continuity of care") OR (TI "continuity of patient care" OR AB "continuity of patient care" OR SU "continuity of patient care") OR (TI "continuum of care" OR AB "continuum of care" OR SU "continuum of care") OR (TI "patient care continuity" OR AB "patient care continuity" OR SU "patient care continuity") OR ((TI discharge OR AB discharge OR SU discharge) N2 (TI plan* OR AB plan* OR SU plan*)) OR ((TI patient? OR AB patient? OR SU patient?) N2 (TI discharge OR AB discharge OR SU discharge)) OR ((TI patient? OR AB patient? OR SU patient?) N2 (TI transfer OR AB transfer OR SU transfer)) OR (TI "transition care*" OR AB "transition care*" OR SU "transition care*") OR (TI "transitional care*" OR AB "transitional care*" OR SU "transitional care*") OR (TI "coordination of care" OR AB "coordination of care" OR SU "coordination of care") OR (TI "co-ordination of care" OR AB "co-ordination of care" OR SU "co-ordination of care") OR (TI "care coordination" OR AB "care coordination" OR SU "care coordination") OR (TI "care co-ordination" OR AB "care co-ordination" OR SU "care co-ordination"))	Expanders - Apply equivalent subjects<br>Search modes - Find all my search terms	Interface - EBSCOhost Research Databases<br>Search Screen - Advanced Search<br>Database - CINAHL
S21	MH "Transitional Care"	Expanders - Apply equivalent subjects<br>Search modes - Find all my search terms	Interface - EBSCOhost Research Databases<br>Search Screen - Advanced Search<br>Database - CINAHL

S20	MH "Patient Transfer"	Expanders - Apply equivalent subjects<br>Search modes - SmartText Searching	Interface - EBSCOhost Research Databases<br>Search Screen - Advanced Search<br>Database - CINAHL
S19	MH "Patient Handoff"	Expanders - Apply equivalent subjects<br>Search modes - SmartText Searching	Interface - EBSCOhost Research Databases<br>Search Screen - Advanced Search<br>Database - CINAHL
S18	MH "Patient Discharge"	Expanders - Apply equivalent subjects<br>Search modes - Find all my search terms	Interface - EBSCOhost Research Databases<br>Search Screen - Advanced Search<br>Database - CINAHL
S17	MH "Continuity of Patient Care"	Expanders - Apply equivalent subjects<br>Search modes - Find all my search terms	Interface - EBSCOhost Research Databases<br>Search Screen - Advanced Search<br>Database - CINAHL
S16	((TI Predictor* OR AB Predictor* OR SU Predictor*) OR ((TI prediction OR AB prediction OR SU prediction) N2 (TI model? OR AB model? OR SU model?)) OR (TI "decision making" OR AB "decision making" OR SU "decision making") OR (TI "decision support" OR AB "decision support" OR SU "decision support"))	Expanders - Apply equivalent subjects<br>Search modes - Find all my search terms	Interface - EBSCOhost Research Databases<br>Search Screen - Advanced Search<br>Database - CINAHL
S15	(MH Forecasting)	Expanders - Apply equivalent subjects<br>Search modes - Find all my search terms	Interface - EBSCOhost Research Databases<br>Search Screen - Advanced Search<br>Database - CINAHL
S14	S4 OR S5 OR S6 OR S7 OR S8 OR S9 OR S10 OR S11 OR S12 OR S13	Expanders - Apply equivalent subjects<br>Search modes - Find all my search terms	Interface - EBSCOhost Research Databases<br>Search Screen - Advanced Search<br>Database - CINAHL
S13	((TI "geriatric assessment?" OR AB "geriatric assessment?" OR SU "geriatric assessment?") OR (TI "nursing assessment?" OR AB "nursing assessment?" OR SU "nursing assessment?"))	Expanders - Apply equivalent subjects<br>Search modes - Find all my search terms	Interface - EBSCOhost Research Databases<br>Search Screen - Advanced Search<br>Database - CINAHL
S12	(MH "Nursing Assessment")	Expanders - Apply equivalent subjects	Interface - EBSCOhost Research Databases<br>Search Screen - Advanced

		Search modes - Find all my search terms	Search<br>Database - CINAHL
S11	(MH "Geriatric Assessment")	Expanders - Apply equivalent subjects<br>Search modes - Find all my search terms	Interface - EBSCOhost Research Databases<br>Search Screen - Advanced Search<br>Database - CINAHL
S10	(TI geriatric* OR TI gerontolog*) OR OR (AB geriatric* OR AB gerontolog*)	Expanders - Apply equivalent subjects<br>Search modes - Find all my search terms	Interface - EBSCOhost Research Databases<br>Search Screen - Advanced Search<br>Database - CINAHL
S9	((TI "geriatric rehabilitation" OR AB "geriatric rehabilitation" OR SU "geriatric rehabilitation") OR ((TI rehabilitation* OR AB rehabilitation* OR SU rehabilitation*) N3 ((TI old* OR AB old* OR SU old*) OR (TI age* OR AB age* OR SU age*))))	Expanders - Apply equivalent subjects<br>Search modes - Find all my search terms	Interface - EBSCOhost Research Databases<br>Search Screen - Advanced Search<br>Database - CINAHL
S8	(MH Geriatrics)	Expanders - Apply equivalent subjects<br>Search modes - Find all my search terms	Interface - EBSCOhost Research Databases<br>Search Screen - Advanced Search<br>Database - CINAHL
S7	((TI "Trochanteric Fracture?" OR AB "Trochanteric Fracture?" OR SU "Trochanteric Fracture?") OR (TI "Subtrochanteric Fracture?" OR AB "Subtrochanteric Fracture?" OR SU "Subtrochanteric Fracture?") OR (TI "Intertrochanteric Fracture?" OR AB "Intertrochanteric Fracture?" OR SU "Intertrochanteric Fracture?") OR (TI "hip fracture?" OR AB "hip fracture?" OR SU "hip fracture?") OR (TI "femoral neck fracture?" OR AB "femoral neck fracture?" OR SU "femoral neck fracture?") OR (TI "osteoporotic fracture?" OR AB "osteoporotic fracture?" OR SU "osteoporotic fracture?") OR (TI orthogeriatric* OR AB orthogeriatric* OR SU orthogeriatric*) OR (TI ortho-geriatric* OR AB ortho-geriatric* OR SU ortho-geriatric*) OR ((TI surgical* OR AB surgical* OR SU surgical*) N3 (TI old* OR AB old* OR SU old*)))	Expanders - Apply equivalent subjects<br>Search modes - Find all my search terms	Interface - EBSCOhost Research Databases<br>Search Screen - Advanced Search<br>Database - CINAHL
S6	S4 OR S5	Expanders - Apply equivalent subjects<br>Search modes - Find all my search terms	Interface - EBSCOhost Research Databases<br>Search Screen - Advanced Search<br>Database - CINAHL

S5	(MH "Hip Fractures+")	Expanders - Apply equivalent subjects<br>Search modes - Find all my search terms	Interface - EBSCOhost Research Databases<br>Search Screen - Advanced Search<br>Database - CINAHL
S4	(MH "Osteoporotic Fractures")	Expanders - Apply equivalent subjects<br>Search modes - Find all my search terms	Interface - EBSCOhost Research Databases<br>Search Screen - Advanced Search<br>Database - CINAHL
S3	S1 OR S2	Expanders - Apply equivalent subjects<br>Search modes - Find all my search terms	Interface - EBSCOhost Research Databases<br>Search Screen - Advanced Search<br>Database - CINAHL
S2	(((TI old* OR AB old* OR SU old*) N3 ((TI person? OR AB person? OR SU person?) OR (TI "people subject?" OR AB "people subject?" OR SU "people subject?") OR (TI patient* OR AB patient* OR SU patient*) OR (TI participant? OR AB participant? OR SU participant?) OR (TI adult? OR AB adult? OR SU adult?))) OR ((TI old* OR AB old* OR SU old*) N2 (TI age* OR AB age* OR SU age*)) OR (TI senior? OR AB senior? OR SU senior?))	Expanders - Apply equivalent subjects<br>Search modes - Find all my search terms	Interface - EBSCOhost Research Databases<br>Search Screen - Advanced Search<br>Database - CINAHL
S1	(MH Aged+)	Expanders - Apply equivalent subjects<br>Search modes - Find all my search terms	Interface - EBSCOhost Research Databases<br>Search Screen - Advanced Search<br>Database - CINAHL

Ovid Emcare

# on 22 February 22	Search
1	exp Aged/
2	((old* adj3 (person$ or people subject$ or patient* or participant$ or adult$)) or (old* adj2 age*) or senior$).ti,ab,kw.
3	1 or 2
4	Osteoporotic Fractures/ or exp Hip Fractures/
5	(Trochanteric Fracture$ or Subtrochanteric Fracture$ or Intertrochanteric Fracture$ or hip fracture$ or femoral neck fracture$ or osteoporotic fracture$ or orthogeriatric* or ortho-geriatric* or (surgical* adj3 old*)).ti,ab,kw.
6	Geriatrics/
7	(geriatric rehabilitation or (rehabilitation* adj3 (old* or age*))).ti,ab,kw.
8	(geriatric* or gerontolog*).ti,kf. or (geriatric* or gerontolog*).ab.<br>/freq=2
9	Geriatric Assessment/

10	Nursing Assessment/
11	(geriatric assessment$ or nursing assessment$).ti,ab,kw.
12	4 or 5 or 6 or 7 or 8 or 9 or 10 or 11
13	Forecasting/
14	(Predictor* or (prediction adj2 model$) or decision making or decision support).ti,kf. or (Predictor* or (prediction adj2 model$) or decision making or decision support).ab. /freq=3
15	"Continuity of Patient Care"/ or Patient Discharge/ or Patient Handoff/ or Patient Transfer/ or Transitional Care/
16	(care continuity or care continuum or continuity of care or continuity of patient care or continuum of care or patient care continuity or (discharge adj2 plan*) or (patient$ adj2 discharge) or (patient$ adj2 transfer) or transition care* or transitional care* or coordination of care or co-ordination of care or care coordination or care co-ordination).ti,ab,kw.
17	Case Management/
18	(case management or care pathway or integrated care pathway or care plan or care planning).ti,ab,kw.
19	"Delivery of Health Care, Integrated"/
20	((delivery adj2 integrated health care) or integrated delivery system* or integrated health care system*).ti,ab,kw.
21	Eligibility Determination/
22	((eligibility adj3 old*) or (eligibility adj3 age*) or (determin* adj3 eligibility) or (readiness adj3 old*) or (readiness adj3 age*)).ti,ab,kw.
23	(rehab* admission$ or rehab* eligibilit* or rehab* potential* or rehab* assessment$).ti,ab,kw.
24	13 or 14 or 15 or 16 or 17 or 18 or 19 or 20 or 21 or 22 or 23
25	3 and 12 and 24
26	(letter or comment or editorial).pt.
27	25 not 26
