# Supplementary material for: Identifying Key Predictors of Appropriate Discharge Destinations for Older Inpatients in Acute Care: Scoping Review
Source: Interact J Med Res. 2026 Jan 22;15:e76582. doi: 10.2196/76582 (PMC12826578; doi:10.2196/76582)
Supplement: Multimedia Appendix 2 [file ijmr-v15-e76582-s002.pdf]

Appendix A2-A: Overview of included quantitative studies ordered by year of publication. P = Prospective observational study (e.g. cohort study), R= Retrospective observational study (e.g. population-based study), ES = Expert Survey

Ref No.	Title	Author, year	Country of origin	Study design	Clinical departments	Participants	
						n	Mean age
1	Level of care and complications among geriatric patients discharged from the medical service of a teaching hospital.	Davis at al., 1984	United States	R	Cardiology; Other: Intensive care, Respiratory care	233	
2	Physicians' outcome predictions for elderly patients. Survival, hospital discharge, and length of stay in a department of internal medicine	Asberg, 1986	Sweden	ES	Internal/general medicine	156	
3	Predictors of immediate and 6-month outcomes in hospitalized elderly patients. The importance of functional status	Narain et al., 1988	United States	P	Cardiology; Gastroenterology; Oncology; Neurology; Pulmonology	396	76.9
4	Self-report versus medical record functional status.	Burns et al., 1992	United States	R	All departments	2504	78
5	Co-ordinating geriatric and general medical services; experience of a geriatric assessment ward in the Royal Infirmary of Edinburgh.	MacLennan et al., 1992	United Kingdom	P	Internal/general medicine	376	81.3
6	Psychosocial predictors of stroke outcomes in an elderly population.	Colantonio et al., 1993	United States	P	Neurology	127	76.7
7	Stroke in patients aged over 75 years: Outcome and predictors	Kalra et al., 1993	United Kingdom	P	Geriatrics; Neurology	96	81.3
8	Predictors of formal home health care use in elderly patients after hospitalization.	Solomon et al., 1993	United States	P	All departments	226	78.8
9	Evaluation of the prognostic value of the health assessment form among patients clinically ready for discharge.	Johansson et al., 1994	Sweden	P	Trauma/Orthogeriatrics; Urology; General/visceral surgery; Vascular surgery	53	82.8
10	Returning home after acute hospitalization in two French teaching hospitals: predictive value of patients' and relatives' wishes.	Zureik et al., 1995	France	P	Emergency department; All departments	417	84.3
11	Hospital admission risk profile (HARP): identifying older patients at risk for functional decline following acute medical illness and hospitalization.	Sager et al., 1996	United States	P	Internal/general medicine	827	79.46
12	Posthospital setting, resource utilization, and self-care outcome in older women with hip fracture	Levi, 1997	United States	P	Trauma/Orthogeriatrics	130	
13	New walking dependence associated with hospitalization for acute medical illness: incidence and significance.	Mahoney et al., 1998	United States	P	All departments	1181	79
14	Predictive validity of the BRASS index in screening patients with post-discharge problems. Blaylock Risk Assessment Screening Score.	Mistiaen et al., 1999	Netherlands	P	All departments	503	76.3
15	Predicting place of discharge from hospital for patients with a stroke or hip fracture on admission.	Bond et al., 2000	United Kingdom	P	Trauma/Orthogeriatrics; Neurology	1012	
16	Factors associated with referral of elderly individuals with cardiac and pulmonary disorders for home care services following hospital discharge.	Narsavage and Naylor, 2000	United States	P	Cardiology; Internal/general medicine; Pulmonology	159	76.124
17	Does the mini nutritional assessment predict hospitalization outcomes in older people?.	van Nes et al., 2001	Switzerland	P	Geriatrics	4677	84.2

18	Characteristics and outcomes of serious traumatic injury in older adults.	Shyu and Lee, 2002	United States	R	Trauma/Orthogeriatrics	38707	77.5
19	Predictors of nursing home placement and home nursing services utilization by elderly patients after hospital discharge in Taiwan.	Richmond et al., 2002	Taiwan	P	Internal/general medicine	233	76.2
20	Functional outcomes in octogenarian trauma	Grossman et al., 2003	United States	R	Trauma/Orthogeriatrics	43297	
21	Predictors of discharge to a skilled nursing facility following hip fracture surgery in New York State.	Aharonoff et al., 2004	United States	R	Trauma/Orthogeriatrics	89723	
22	Delirium symptoms and low dietary intake in older inpatients are independent predictors of institutionalization: a 1-year prospective population-based study.	Bourdel-Marchasson et al., 2004	France	P	Geriatrics	427	84.87
23	Prevalence and outcomes of low mobility in hospitalized older patients.	Brown et al., 2004	United States	P	All departments	498	78.7
24	Co-morbidity in an elderly inpatient population: Are the risks of co-existing conditions differentially distributed?	Broyles et al., 2005	United States	R	All departments	167738	
25	Gender influences on burn outcomes in the elderly	Chang et al., 2005	United States	R	Other: Burn center	94	76.1
26	Racial differences in institutionalization after hip fractures: California hospital discharge data.	Ganesan et al., 2005	United States	R	Trauma/Orthogeriatrics	324760	
27	Vital capacity as a predictor of outcome in elderly patients with rib fractures.	Bakhos et al., 2006	United States	R	Trauma/Orthogeriatrics	38	80.2
28	Prediction of postoperative morbidity, mortality and rehabilitation in hip fracture patients: The cumulated ambulation score	Foss et al., 2006	Denmark	P	Trauma/Orthogeriatrics	426	
29	Healthcare utilization of elderly persons hospitalized after a noninjurious fall in a Swiss academic medical center	Seematter-Bagnoud et al., 2006	Switzerland	P	Geriatrics; Internal/general medicine; Emergency department	690	82.4
30	Upper extremity function, an independent predictor of adverse events in hospitalized elderly	Abizanda et al., 2007	Spain	P	Geriatrics	356	83.1
31	Prognostic implications of hyponatremia in elderly hospitalized patients	Chua et al., 2007	United Kingdom	R	Geriatrics	103	82
32	Bouncing back: patterns and predictors of complicated transitions 30 days after hospitalization for acute ischemic stroke.	Kind et al., 2007	United States	R	Neurology	39384	79.6
33	Geriatric syndromes as predictors of adverse outcomes of hospitalization.	Anpalahan and Gibson, 2008	Australia	P	Internal/general medicine	110	83.29
34	Does depression predict adverse outcomes for older medical inpatients? A prospective cohort study of individuals screened for a trial*	Cullum et al., 2008	United Kingdom	P	Internal/general medicine	617	
35	Elevated serum cardiac troponin I in older patients with hip fracture: incidence and prognostic significance.	Fisher et al., 2008	Australia	P	Trauma/Orthogeriatrics	238	81.9
36	Rehabilitation and nursing home admission after hospitalization in acute geriatric patients.	Marengoni et al., 2008	Italy	P	Geriatrics	830	78.5
37	Emergent and elective colon surgery in the extreme elderly: Do the results warrant the operation?	Morse et al., 2008	United States	R	General/visceral surgery	104	84.125

38	The aftermath of hip fracture: discharge placement, functional status change, and mortality.	Bentler et al., 2009	United States	R	Trauma/Orthogeriatrics	495	85
39	Factors identified by experts to support decision making for post acute referral.	Bowles et al., 2009	United States	ES	All departments	355	74.25
40	Frailty is associated with postoperative complications in older adults with medical problems.	Dasgupta et al., 2009	Canada	P	Trauma/Orthogeriatrics; Neurosurgery; General/visceral surgery; Vascular surgery	125	77
41	Does dementia predict adverse hospitalization outcomes? A prospective study in aged inpatients	Zekry et al., 2009	Switzerland	P	Geriatrics	435	85.3
42	Characteristics and outcomes of injured older adults after hospital admission	Aitken et al., 2010	Australia	R	Trauma/Orthogeriatrics	6069	
43	Survey of geriatricians on the effect of fecal incontinence on nursing home referral.	Grover et al., 2010	United States	ES	Geriatrics		
44	Functional decline two weeks before hospitalization in an elderly population.	Isaia et al., 2010	Italy	P	Geriatrics	123	82.2
45	Institutional variations in frequency of discharge of elderly intensive care survivors to postacute care facilities.	Kramer and Zimmerman, 2010	United States	P	Cardiology; Other: Intensive care unit	13370	
46	Frailty as a predictor of surgical outcomes in older patients.	Makary et al., 2010	United States	P	All departments	594	72.82
47	Geriatrics index of comorbidity was the most accurate predictor of death in geriatric hospital among six comorbidity scores.	Zekry et al., 2010	Switzerland	P	Geriatrics	444	85.3
48	Components of geriatric assessments predict thoracic surgery outcomes	Kothari et al., 2011	United States	P	Other: Thoracic oncology surgery	60	76.73
49	Accumulated frailty characteristics predict postoperative discharge institutionalization in the geriatric patient.	Robinson et al., 2011	United States	P	Urology; General/visceral surgery; Vascular surgery; Cardiac surgery,Thoracic surgery	223	73
50	Identification of factors associated with a need for rehabilitation for patients over 65 years following admission to a general and vascular surgical service.	Smyth and Connor, 2011	New Zealand	R	General/visceral surgery; Vascular surgery	2632	
51	Outcome in hip fracture patients related to anemia at admission and allogeneic blood transfusion: An analysis of 1262 surgically treated patients	Vochteloo et al., 2011	Netherlands	R+P	Trauma/Orthogeriatrics	1262	83.6
52	Exploring predictors of complication in older surgical patients: a deficit accumulation index and the Braden Scale.	Cohen et al., 2012	United States	R	General/visceral surgery	102	72.2
53	Efficacy of early clinical evaluation in predicting direct home discharge of elderly patients after hospitalization in internal medicine.	Gambier et al., 2012	France	P	Internal/general medicine	97	86
54	Patient disposition and long-term outcomes after valve surgery in octogenarians.	Henry et al., 2012	United States	P	Vascular surgery	307	82.9
55	Most patients regain prefracture basic mobility after hip fracture surgery in a fast-track programme.	Kristensen and Kehlet, 2012	Denmark	P	Trauma/Orthogeriatrics	213	
56	Impact of psychotic disorders on discharge dispositions of adults 65 or older after a general medical inpatient stay	Nath and Marcus, 2012	United States	R	All departments	2334130	
57	Assessing gait speed in acutely ill older patients admitted to an acute care for elders hospital unit.	Ostir et al., 2012	United States	P	Geriatrics	322	76.1

58	Risk factors for failure to return to the pre-fracture place of residence after hip fracture: a prospective longitudinal study of 444 patients.	Vochteloo et al., 2012	Netherlands	P	Trauma/Orthogeriatrics	444	83.4
59	Comprehensive geriatric assessment of risk factors associated with adverse outcomes and resource utilization in cancer patients undergoing abdominal surgery.	Badgwell et al., 2013	United States	P	General/visceral surgery	111	
60	Relationship between asking an older adult about falls and surgical outcomes	Jones et al., 2013	United States	P	General/visceral surgery; Other: Cardiac surgery	235	74
61	The Higher Care At Discharge Index (HCDI): identifying older patients at risk of requiring a higher level of care at discharge.	Lakhan et al., 2013	Australia	P	Internal/general medicine; Emergency department	360	81.8
62	Outcomes of early delirium diagnosis after general anesthesia in the elderly	Neufeld et al., 2013	United States	P	Trauma/Orthogeriatrics; Urology; General/visceral surgery; Gynaecology	91	79
63	Utilizing multiple methods to classify malnutrition among elderly patients admitted to the medical and surgical intensive care units (ICU)	Sheean et al., 2013	United States	P	All departments	260	74.26
64	Do clinical characteristics and outcome in nonagenarians with a hip fracture differ from younger patients?	Vochteloo et al., 2013	Netherlands	R+P	Trauma/Orthogeriatrics	1244	83.56
65	Geriatric Syndromes Predict Postdischarge Outcomes Among Older Emergency Department Patients: Findings From the interRAI Multinational Emergency Department Study	Costa et al., 2014	Canada	P	Emergency department	1436	
66	A multicomponent approach to identify predictors of hospital outcomes in older in-patients: a multicentre, observational study.	De Buyser et al., 2014	Italy	P	Geriatrics; Internal/general medicine	1123	81.5
67	Psychosocial factors modify the association of frailty with adverse outcomes: a prospective study of hospitalised older people.	Dent and Hoogendeijk, 2014	Australia	P	Geriatrics	172	85.2
68	Medication-related factors affecting discharge to home.	Hashimoto et al., 2014	Japan	R	All departments	282	78.7
69	Superiority of frailty over age in predicting outcomes among geriatric trauma patients: a prospective analysis.	Joseph et al., 2014	United States	P	Trauma/Orthogeriatrics	250	77.9
70	Multidimensional Frailty Score for the Prediction of Postoperative Mortality Risk	Kim et al., 2014	South Korea	P	General/visceral surgery	275	75.42
71	Use and validation of the Balance Outcome Measure for Elder Rehabilitation in acute care.	Kuys et al., 2014	Australia	P	Internal/general medicine	44	77
72	A Risk Index for Geriatric Acute Medical Admissions (RIGAMA).	Romero-Ortuno et al., 2014	Ireland	R	Internal/general medicine; Emergency department	29982	
73	Can frailty predict complicated care needs and length of stay?.	Rose et al., 2014	Australia	P	Internal/general medicine	133	86.5
74	Effect of frailty on short- and mid-term outcomes in vascular surgical patients	Ambler et al., 2015	United Kingdom	P	Vascular surgery	413	
75	Traumatic brain injury in the elderly: A level 1 trauma centre study.	de Guise et al., 2015	Canada	R	Trauma/Orthogeriatrics	1812	
76	Geriatric Assessment as a Predictor of Delirium and Other Outcomes in Elderly Patients With Cancer.	Korc-Grodzicki et al., 2015	United States	R	Geriatrics; Oncology	416	

77	Perioperative factors predicting poor outcome in elderly patients following emergency general surgery: a multivariate regression analysis.	Lees et al., 2015	Canada	R	General/visceral surgery	257	72
78	Which Patients Require More Care after Hospital Discharge? An Analysis of Post-Acute Care Use among Elderly Patients Undergoing Elective Surgery.	Sacks et al., 2015	United States	R	General/visceral surgery; Vascular surgery	14812	75.64
79	Skilled care utilization after abdominal and pelvic cancer surgery in older patients	Alexander et al., 2016	United States	R	Geriatrics	592	79.98
80	Frailty as a Predictor of Acute Kidney Injury in Hospitalized Elderly Patients: A Single Center, Retrospective Cohort Study.	Baek et al., 2016	South Korea	R	All departments	533	76.3
81	The Effect of an Impaired Arousal on Short- and Long-Term Mortality of Elderly Patients Admitted to an Acute Geriatric Unit.	Bellelli et al., 2016	Italy	P	Geriatrics	2477	84.2
82	Quantitative increase in frailty is associated with diminished survival after transcatheter aortic valve replacement.	Chauhan et al., 2016	United States	R	Cardiology; Vascular surgery	342	81.85
83	Comparison of Frailty Measures as Predictors of Outcomes After Orthopedic Surgery.	Cooper et al., 2016	United States	P	Trauma/Orthogeriatrics	415	76.8
84	Scoliosis surgery in the elderly: Complications, readmissions, reoperations and mortality	Drazin et al., 2016	United States	R	Neurosurgery	453	73.8
85	Assessment of mortality risk in elderly persons with obstructive sleep apnea diagnosed with pneumonia	Gibson et al., 2016	United States	R	Internal/general medicine	1768185	
86	Creation of a decision aid for goal setting after geriatric burns: a study from the prognostic assessment of life and limitations after trauma in the elderly [PALLIATE] consortium.	Hodgman et al., 2016	United States	R	Other: Burn center	8001	
87	Self-reported mobility in older patients predicts early postoperative outcomes after elective noncardiac surgery	Kim et al., 2016	United States	P	Trauma/Orthogeriatrics; Neurosurgery; General/visceral surgery; Gynaecology; Head and neck surgery; Vascular surgery	197	75.2
88	Association Between Hospital Admission Risk Profile Score and Skilled Nursing or Acute Rehabilitation Facility Discharges in Hospitalized Older Adults	Liu et al., 2016	United States	R	Internal/general medicine	428	80.5
89	Frailty and Geriatric Syndromes in Vascular Surgical Ward Patients.	McRae et al., 2016	Australia	P	Vascular surgery	110	75
90	Early Ambulation Decreases Length of Hospital Stay, Perioperative Complications and Improves Functional Outcomes in Elderly Patients Undergoing Surgery for Correction of Adult Degenerative Scoliosis	Adogwa et al., 2017	United States	R	Neurosurgery	125	73.48
91	Comparison of clipping and coiling in elderly patients with unruptured cerebral aneurysms	Bekelis et al., 2017	United States	R	Neurosurgery	8705	72.05
92	A prospective cohort study of older surgical inpatients examining the prevalence and implications of frailty	Cheung et al., 2017	Australia	P	Trauma/Orthogeriatrics; General/visceral surgery; Vascular surgery; Cardiothoracic	100	78
93	FRAIL Questionnaire Screening Tool and Short-Term Outcomes in Geriatric Fracture Patients	Gleason et al., 2017	United States	R	Trauma/Orthogeriatrics; Geriatrics	175	82.3
94	Frailty status at admission to hospital predicts multiple adverse outcomes.	Hubbard et al., 2017	Australia	P	Trauma/Orthogeriatrics; Internal/general medicine;	1418	81

					Urology; General/visceral surgery; Vascular surgery		
95	Spinal Fractures in Older Adult Patients Admitted After Low-Level Falls: 10-Year Incidence and Outcomes	Jawa et al., 2017	United States	R	Trauma/Orthogeriatrics	4202	
96	Redefining the association between old age and poor outcomes after trauma: The impact of frailty syndrome	Joseph et al., 2017	United States	P	Trauma/Orthogeriatrics	350	78.44
97	The impact of Glasgow Coma Scale-age prognosis score on geriatric traumatic brain injury outcomes.	Khan et al., 2017	United States	R	Trauma/Orthogeriatrics	8750	77.8
98	The association of frailty with outcomes and resource use after emergency general surgery: A population-based cohort study	McIsaac et al., 2017	Canada	R	General/visceral surgery	77184	76.77
99	Risk factors associated with residential aged care, respite and transitional aged care admission for older people following an injury-related hospitalisation	Mitchell et al., 2017	Australia	R	Trauma/Orthogeriatrics	191301	80.68
100	Mortality, Geriatric, and Nongeriatric Surgical Risk Factors Among the Eldest Old: A Prospective Observational Study.	Pelavski et al., 2017	Spain	P	Other: Surgery	127	
101	Increasing use of reverse total shoulder arthroplasty for proximal humerus fractures in elderly patients	Rajaee et al., 2017	United States	R	Trauma/Orthogeriatrics	38729	
102	The association of geriatric syndromes with hospital outcomes	Romero-Ortuno et al., 2017	United Kingdom	R	Emergency department	8202	84.1
103	Independent Association Between Preoperative Cognitive Status and Discharge Location After Surgery: A Strategy to Reduce Resource Use After Surgery for Deformity.	Adogwa et al., 2018	United States	P	Neurosurgery	82	73.84
104	Predictors and Sequelae of Postoperative Delirium in Geriatric Hip Fracture Patients	Arshi et al., 2018	United States	R	Trauma/Orthogeriatrics	8439	
105	Determining discharge destination in geriatric evaluation and management units: Is progressive goal attainment a better early indicator of discharge destination than improvement in functional independence measure scores?.	Black et al., 2018	Australia	P	Geriatrics	82	82.4
106	Association of pre-operative medication use with post-surgery mortality and morbidity in oncology patients receiving comprehensive geriatric assessment	Choi et al., 2018	South Korea	R	Other: Cancer surgery	475	
107	Frailty score on admission predicts mortality and discharge disposition in elderly trauma patients over the age of 65 y.	Curtis et al., 2018	United States	R	Trauma/Orthogeriatrics	1403	77.6
108	Prestroke Mobility and Dementia as Predictors of Stroke Outcomes in Patients Over 65 Years of Age: A Cohort Study From The Swedish Dementia and Stroke Registries.	Garcia-Ptacek et al., 2018	Sweden	R	Neurology	9662	
109	Predictors of Dependency in Geriatric Trauma Patients with Rib Fractures: A Population Study.	Halevi et al., 2018	United States	R	Trauma/Orthogeriatrics	16632	
110	Sarcopenia affects conservative treatment of osteoporotic vertebral fracture	Iida et al., 2018	Japan	P	Trauma/Orthogeriatrics	396	81.93

111	Better preoperative physical performance reduces the odds of complication severity and discharge to care facility after abdominal cancer resection in people over the age of 70 - A prospective cohort study.	Karlsson et al., 2018	Sweden	P	General/visceral surgery	197	76
112	The Six-Item Cognitive Impairment Test Is Associated with Adverse Outcomes in Acutely Hospitalized Older Patients: A Prospective Cohort Study.	Lucke et al., 2018	Netherlands	P	Trauma/Orthogeriatrics; Cardiology; Internal/general medicine; Urology; Neurology; General/visceral surgery	1252	
113	Clinical outcomes in older surgical patients with mild cognitive impairment	Racine et al., 2018	United States	P	Trauma/Orthogeriatrics; Neurosurgery; General/visceral surgery; Vascular surgery; elective surgery	560	76.7
114	Measuring Functional Status in Hospitalized Older Adults Through Electronic Health Record Documentation.	Sinvani et al., 2018	United States	R	All departments	2383	84.73
115	Frailty as a predictor of hospital length of stay after elective total joint replacements in elderly patients	Wang et al., 2018	Canada	P	Trauma/Orthogeriatrics	87	
116	The Edmonton Frail Scale Improves the Prediction of 30-Day Mortality in Elderly Patients Undergoing Cardiac Surgery: A Prospective Observational Study	Amabili et al., 2019	Belgium	P	Other: Cardiac surgery	254	
117	Comparison of Geriatric Trauma Outcomes When Admitted to a Medical or Surgical Service After a Fall.	Barry et al., 2019	United States	R	Trauma/Orthogeriatrics; Emergency department	2172	80.76
118	Routine frailty assessment predicts postoperative complications in elderly patients across surgical disciplines - A retrospective observational study	Birkelbach et al., 2019	Germany	R	Trauma/Orthogeriatrics; Urology; General/visceral surgery; Gynaecology; Head and neck surgery; Dermatology	1186	
119	Delirium after hip hemiarthroplasty for proximal femoral fractures in elderly patients: risk factors and clinical outcomes.	de Jong et al., 2019	Netherlands	R	Trauma/Orthogeriatrics	463	81
120	Frailty as a prognostic factor for the critically ill older adult trauma patients	Hamidi et al., 2019	United States	R	Trauma/Orthogeriatrics	34854	76.7
121	Prospective evaluation and comparison of the predictive ability of different frailty scores to predict outcomes in geriatric trauma patients.	Hamidi et al., 2019	United States	P	Trauma/Orthogeriatrics	341	76
122	The Barthel Index and the Cumulated Ambulation Score are superior to the de Morton Mobility Index for the early assessment of outcome in patients with a hip fracture admitted to an acute geriatric ward.	Hulsbaek et al., 2019	Denmark	P	Trauma/Orthogeriatrics	222	
123	Frailty defined by 19 items as a predictor of short-term functional recovery in patients with hip fracture.	Inoue et al., 2019	Japan	R	Trauma/Orthogeriatrics	274	83.7
124	The importance of increased awareness for delirium in elderly patients with rib fractures after blunt chest wall trauma: A retrospective cohort study on risk factors and outcomes	Janssen et al., 2019	Netherlands	R	Trauma/Orthogeriatrics	191	
125	The impact of metabolic syndrome on 30-day outcomes in geriatric hip fracture surgeries.	Malik et al., 2019	United States	R	Trauma/Orthogeriatrics	31621	

126	Derivation and Validation of a Generalizable Preoperative Frailty Index Using Population-based Health Administrative Data	McIsaac et al., 2019	Canada	R	Trauma/Orthogeriatrics; Urology; General/visceral surgery; Vascular surgery	415704	73.86
127	New associations of the Multidimensional Prognostic Index.	Meyer et al., 2019	Germany	P	Internal/general medicine; Other: Nephrology, Rheumatology, Diabetology	135	78.1
128	Relationship of Malnutrition During Hospitalization With Functional Recovery and Postdischarge Destination in Elderly Stroke Patients.	Sato et al., 2019	Japan	R	Neurology; Neurosurgery	205	77
129	Health outcome of older hospitalized patients in internal medicine environments evaluated by Identification of Seniors at Risk (ISAR) screening and geriatric assessment.	Scharf et al., 2019	Germany	P	Cardiology; Gastroenterology; Other: Nephrology	547	78.14
130	Acute Exacerbation of Chronic Obstructive Pulmonary Disease in Oldest Adults: Predictors of In-Hospital Mortality and Need for Post-acute Care.	Spannella et al., 2019	Italy	P	Geriatrics; Internal/general medicine	121	87
131	Assessment of Mobility in Older People Hospitalized for Medical Illness Using the de Morton Mobility Index and Cumulated Ambulation Score-Validity and Minimal Clinical Important Difference.	Trostrup et al., 2019	Denmark	P	Geriatrics; Internal/general medicine	235	84.8
132	Changes in vulnerability among older patients with cardiovascular disease in the first 90 days after hospital discharge: A secondary analysis of a cohort study.	Wang et al., 2019	United States	R	Cardiology	834	
133	Total Hip Arthroplasty Outperforms Hemiarthroplasty in Patients Aged 65 Years and Older: A Propensity-Matched Study of Short-Term Outcomes	Warren et al., 2019	United States	R	Trauma/Orthogeriatrics	5590	76.55
134	Association of depression with malnutrition, grip strength and impaired cognitive function among senior trauma patients.	Wiegand et al., 2019	Switzerland	P	Trauma/Orthogeriatrics	273	79.4
135	Modified frailty index and hypoalbuminemia as predictors of adverse outcomes in older adults presenting to acute general surgical unit.	Abraham et al., 2020	Australia	P	General/visceral surgery	145	77
136	Postacute Care Utilization in Postsurgical Orthogeriatric Hip Fracture Care.	Arshi et al., 2020	United States	R	Trauma/Orthogeriatrics	8133	
137	Geriatric or cardiac rehabilitation? Predictors of treatment pathways in advanced age patients after transcatheter aortic valve implantation.	Eichler et al., 2020	Germany	P	Cardiology	249	80.7
138	Prognostic factors for discharge to home and residing at home 12 months after hip fracture: an Anoia hip study.	Gamboa-Arango et al., 2020	Spain	P	Trauma/Orthogeriatrics; Geriatrics	273	84.8
139	Physical performance among patients aged 70 + in acute care: a preliminar comparison between the Short Physical Performance Battery and the De Morton Mobility Index with regard to sensitivity to change and prediction of discharge destination	Gazzotti et al., 2020	Switzerland	R	Trauma/Orthogeriatrics	69	83.7
140	Age, Frailty, and Comorbidity as Prognostic Factors for Short-Term Outcomes in Patients With Coronavirus Disease 2019 in Geriatric Care	Hagg et al., 2020	Sweden	P	Geriatrics	967	82.32
141	Associations of 4AT with mobility, length of stay and mortality in hospital and discharge destination among patients admitted with hip fractures.	Lisk et al., 2020	United Kingdom	P	Trauma/Orthogeriatrics	537	83.7
142	Safety of Major Abdominal Operations in the Elderly: A Study of Geriatric-Specific Determinants of Health.	Martin et al., 2020	United States	R	General/visceral surgery	4165	

143	Frailty as a Predictor of Death or New Disability After Surgery: A Prospective Cohort Study.	McIsaac et al., 2020	Canada	P	Trauma/Orthogeriatrics; Neurosurgery; General/visceral surgery; Vascular surgery; Other: Thoracic surgery	702	73.4
144	Body mass index and recovery of activities of daily living in older patients with femoral fracture: An analysis of a national inpatient database in Japan	Nishioka et al., 2020	Japan	R	Trauma/Orthogeriatrics	13348	
145	[Challenges of discharge management in geriatric traumatology : Example of an integrated orthogeriatric service].	Scheidt et al., 2020	Germany	R	Trauma/Orthogeriatrics	312	80.95
146	Brief Preoperative Screening for Frailty and Cognitive Impairment Predicts Delirium after Spine Surgery.	Susano et al., 2020	United States	P	Neurosurgery	219	
147	Association between Physical Activity Levels in the Hospital Setting and Hospital-Acquired Functional Decline in Elderly Patients	Tasheva et al., 2020	Switzerland	P	Internal/general medicine	177	
148	The 5 and 11 Factor Modified Frailty Indices are Equally Effective at Outcome Prediction Using TQIP	Tracy et al., 2020	United States	R	Trauma/Orthogeriatrics	8467	
149	Outcomes of vulnerable elderly patients undergoing elective major surgery: a prospective cohort study.	Wang et al., 2020	Canada	P	Trauma/Orthogeriatrics; General/visceral surgery; Vascular surgery	270	
150	Frailty screening in hospitalised older adults: How does the brief Dutch National Safety Management Program perform compared to a more extensive approach?	Warnier et al., 2020	Netherlands	R	All departments	2573	78.8
151	Evaluation of Postoperative Functional Health Status Decline Among Older Adults.	Zhang et al., 2020	United States	R	Other: Surgical departments	2013	84.9
152	A Fall Within 3 Months Before Total Joint Arthroplasty is Associated With Adverse Outcomes in Elderly Patients.	Chaudhry et al., 2021	United States	R	Trauma/Orthogeriatrics	9865	
153	Prediction of Postoperative Outcomes Following Hip Fracture Surgery: Independent Validation and Recalibration of the Nottingham Hip Fracture Score.	Doherty et al., 2021	United Kingdom	P	Trauma/Orthogeriatrics	3092	82.7
154	Complications and Discharge after Radical Cystectomy for Older Patients with Muscle-Invasive Bladder Cancer: The ELCAPA-27 Cohort Study.	Geiss et al., 2021	France	P	Geriatrics; Urology	62	
155	Preoperative functional status is associated with discharge to nonhome in geriatric individuals.	Hung et al., 2021	United States	R	Other: Surgical department	44219	
156	Low muscle mass affect hip fracture treatment outcomes in older individuals: a single-institution case-control study	Iida et al., 2021	Japan	P	Trauma/Orthogeriatrics	337	84.1
157	Preoperative cognitive impairment as a predictor of postoperative outcomes in elderly patients undergoing spinal surgery for degenerative spinal disease	Kim et al., 2021	South Korea	P	Neurosurgery	122	71.62
158	Frequency of hypertension and renal insufficiency and their clinical significance for the success of patients in early rehabilitative geriatric complex treatment	Luders et al., 2021	Germany	R	Geriatrics	197	82.6
159	Analyzing Outcomes Among Older Adults With Necrotizing Soft-Tissue Infections in the United States.	McCarty et al., 2021	United States	R	General/visceral surgery	1427	75.4

160	Preoperative frailty assessment with the Robinson Frailty Score, Edmonton Frail Scale, and G8 and adverse postoperative outcomes in older surgical patients with cancer.	Nishijima et al., 2021	Japan	P	Geriatrics; Oncology; General/visceral surgery; Gynaecology; Head and neck surgery	114	
161	[Acute geriatric treatment of older trauma patients : Influence on mobility, autonomy and postdischarge destination].	Palzer et al., 2021	Switzerland	P	Trauma/Orthogeriatrics	164	83.3
162	Classification and assessment of medication risk in the elderly (Care): Use of a medication risk score to inform patients' readmission likelihood after hospital discharge	Sanfilippo et al., 2021	United States	R	All departments	1386	80
163	Impact of concomitant injuries in geriatric patients with proximal femur fracture : an analysis of the Registry for Geriatric Trauma.	Schoeneberg et al., 2021	Germany	R	Trauma/Orthogeriatrics	22602	
164	Does the frailty index predict discharge disposition and length of stay at the hospital and rehabilitation facilities?.	Schuijt et al., 2021	United States	R	Trauma/Orthogeriatrics	313	83.83
165	Distal femur fractures have a higher mortality rate compared to hip fractures among the elderly: Insights from the National Trauma Data Bank.	Tsai et al., 2021	United States	R	Trauma/Orthogeriatrics	259538	79.8
166	Early Predictors for Discharge to Geriatric Rehabilitation after Hip Fracture Treatment of Older Patients.	van Dartel et al., 2021	Netherlands	R	Trauma/Orthogeriatrics	21176	83.2
167	Factors for Increased Hospital Stay and Utilization of Post -Acute Care Facilities in Geriatric Orthopaedic Fracture Patients.	Van Der Vliet et al., 2021	United States	R	Trauma/Orthogeriatrics	1074	
168	The DEPARTS Score: A Novel Tool for Predicting Discharge Disposition in Geriatric Trauma Patients	Yeates et al., 2021	United States	R	Trauma/Orthogeriatrics	132956	
169	Is the Parker Mobility Score in the older patient with a traumatic hip fracture associated with discharge disposition after surgery? A retrospective cohort study.	Kusen et al., 2022	Netherlands	R	Trauma/Orthogeriatrics	649	
170	Early predictors of discharge to home among severely injured geriatric patients: A single-system retrospective cohort study	Lai et al., 2022	Canada	R	Trauma/Orthogeriatrics	1548	
171	Understanding the role of informal caregivers in postoperative care transitions for older patients.	Sokas et al., 2022	United States	R	Trauma/Orthogeriatrics; Urology; General/visceral surgery; Gynaecology; Head and neck surgery; Vascular surgery; Cardiac surgery; Plastic surgery; Thoracic surgery	18494	73.7

Appendix A2-B: Overview of included qualitative studies ordered by year of publication.

<table>
<tr><th rowspan="2">Ref No.</th><th rowspan="2">Title</th><th rowspan="2">Author, year</th><th rowspan="2">Country</th><th rowspan="2">Clinical department</th><th rowspan="2">Method(s)</th><th colspan="3">Participants</th></tr>
<tr><th>Group(s)</th><th>n (patients)</th><th>n (total)</th></tr>
<tr><td>172</td><td>Experiences of elderly patients concerning discharge from hospital</td><td>Tierney et al., 1993</td><td>United Kingdom</td><td>Trauma/Orthogeriatrics; Geriatrics; Internal/general medicine; General/visceral surgery; Ophtalmology</td><td>Interviews</td><td>Patients</td><td colspan="2">34</td></tr>
<tr><td>173</td><td>Patients' and professionals' perceptions of quality in discharge planning</td><td>Bull, 1994</td><td rowspan="2">United States</td><td rowspan="2">Internal/general medicine</td><td>Interviews</td><td rowspan="2">Patients, physicians, nurses, social workers</td><td rowspan="2">25</td><td rowspan="2">63</td></tr>
<tr><td>174</td><td>A discharge planning questionnaire for clinical practice.</td><td>Bull, 1994</td><td>Interviews, validation of an instrument</td></tr>
<tr><td>175</td><td>Managing the incongruities: the hospital discharge experience for elderly patients, their families, and nurses.</td><td>Congdon, 1994</td><td>United States</td><td>Trauma/Orthogeriatrics</td><td>Interviews, Observation, field notes, review of patient records</td><td>Patients, relatives, nurses</td><td>8</td><td>24</td></tr>
<tr><td>176</td><td>Managing Patient Discharge to Home: The Challenges of Achieving Quality of Care</td><td>McWilliam and Sangster, 1994</td><td>Canada</td><td>All departments</td><td>Interviews, Observation, review of patient records</td><td>Patients, formal caregivers, informal caregivers, policy experts</td><td>21</td><td>198</td></tr>
<tr><td>177</td><td>Gaps in Discharge Planning</td><td>Bull and Kane, 1996</td><td>United States</td><td>Internal/general medicine</td><td>Interviews</td><td>Patients, relatives, physicians, nurses, social workers</td><td>25</td><td>316</td></tr>
<tr><td>178</td><td>Development and testing of a measure designed to assess the quality of care transitions</td><td>Coleman et al., 2002</td><td>United States</td><td>All departments</td><td>Group sessions, validation of an instrument</td><td>Patients, informal caregivers</td><td>na</td><td>49</td></tr>
<tr><td>179</td><td>How Hospital Clinicians Select Patients for Skilled Nursing Facilities</td><td>Burke et al., 2017</td><td>United States</td><td>Trauma/Orthogeriatrics; Geriatrics; Internal/general medicine</td><td>Interviews</td><td>Physicians, nurses, physiotherapists, occupational therapists, social workers, case managers</td><td></td><td>25</td></tr>
<tr><td>180</td><td>Views from health professionals on accessing rehabilitation for people with dementia following a hip fracture</td><td>Isbel and Jamieson, 2017</td><td>Australia</td><td>Trauma/Orthogeriatrics</td><td>Interviews</td><td>Physicians, nurses, physiotherapists</td><td></td><td>12</td></tr>
<tr><td>181</td><td>Exploring how occupational therapists and physiotherapists evaluate rehabilitation potential of older people in acute care</td><td>Bradley et al., 2022</td><td>United Kingdom</td><td>Geriatrics</td><td>Interviews, observation, review of patient records</td><td>Patients, physiotherapists, occupational therapists</td><td>5</td><td>10</td></tr>
</table>

References

1. Davis JW, Shapiro MF, Kane RL. Level of care and complications among geriatric patients discharged from the medical service of a teaching hospital. Journal of the American Geriatrics Society United States; 1984;32(6):427–30.

2. Åsberg KH. Physicians' Outcome Predictions for Elderly Patients: Survival, Hospital Discharge, and Length of Stay in a Department of Internal Medicine. Scandinavian Journal of Social Medicine 1986 Sep;14(3):127–132. doi: 10.1177/140349488601400304

3. Narain P., Rubenstein L.Z., Wieland D., Rosbrook B., Strome L.S., Pietruszka F., Morley J.E. Predictors of immediate and 6-month outcomes in hospitalized elderly patients. The importance of functional status. Journal of the American Geriatrics Society United States: Blackwell Publishing Inc. (350 Main Street, Malden MA 02148, United States); 1988;36(9):775–783.

4. Burns RB, Moskowitz MA, Ash A, Kane RL, Finch MD, Bak SM. Self-report versus medical record functional status. Medical care United States; 1992;30(5 Suppl):MS85-95.

5. MacLennan WJ, Chapman BJ, Smith M, Prescott RJ, Wang JX. Co-ordinating geriatric and general medical services; experience of a geriatric assessment ward in the Royal Infirmary of Edinburgh. Scottish medical journal Scotland; 1992;37(3):80–2.

6. Colantonio A, Kasl SV, Ostfeld AM, Berkman LF. Psychosocial predictors of stroke outcomes in an elderly population. Journal of gerontology United States; 1993;48(5):S261-8.

7. Kalra L., Smith D.H., Crome P. Stroke in patients aged over 75 years: Outcome and predictors. Postgraduate Medical Journal United Kingdom: BMJ Publishing Group (Tavistock Square, London WC1H 9JR, United Kingdom); 1993;69(807):33–36.

8. Solomon DH, Wagner DR, Marenberg ME, Acampora D, Cooney LMJ, Inouye SK. Predictors of formal home health care use in elderly patients after hospitalization. Journal of the American Geriatrics Society United States; 1993;41(9):961–6.

9. Johansson I, Hamrin E, Larsson G. Evaluation of the prognostic value of the health assessment form among patients clinically ready for discharge. Journal of nursing management England; 1994;2(2):77–85.

10. Zureik M, Lang T, Trouillet JL, Davido A, Tran B, Levy A, Lombrail P. Returning home after acute hospitalization in two French teaching hospitals: predictive value of patients' and relatives' wishes. Age and ageing England; 1995;24(3):227–34.

11. Sager MA, Rudberg MA, Jalaluddin M, Franke T, Inouye SK, Landefeld CS, Siebens H, Winograd CH. Hospital admission risk profile (HARP): identifying older patients at risk for functional decline following acute medical illness and hospitalization. Journal of the American Geriatrics Society United States; 1996;44(3):251–7.

12. Levi S.J. Posthospital setting, resource utilization, and self-care outcome in older women with hip fracture. Archives of Physical Medicine and Rehabilitation United States: W.B. Saunders (Independence Square West, Philadelphia PA 19106-3399, United States); 1997;78(9):973–979. doi: 10.1016/S0003-9993%2897%2990060-5

13. Mahoney JE, Sager MA, Jalaluddin M. New walking dependence associated with hospitalization for acute medical illness: incidence and significance. Journals of Gerontology Series A: Biological Sciences & Medical Sciences Oxford University Press / USA; 1998;53A(4):M307–M312. doi: 10.1093/gerona/53a.4.m307

14. Mistiaen P, Duijnhouwer E, Prins-Hoekstra A, Ros W, Blaylock A. Predictive validity of the BRASS index in screening patients with post-discharge problems. Blaylock Risk Assessment Screening Score. Journal of advanced nursing England; 1999;30(5):1050–6.

15. Bond J, Gregson B, Lecouturier MSJ, Rousseau N, Rodgers H. Predicting Place of Discharge from Hospital for Patients with a Stroke or Hip Fracture on Admission. J Health Serv Res Policy 2000 Jul;5(3):133–139. doi: 10.1177/135581960000500303

16. Narsavage GL, Naylor MD. Factors associated with referral of elderly individuals with cardiac and pulmonary disorders for home care services following hospital discharge. Journal of gerontological nursing United States; 2000;26(5):14–20.

17. Van Nes MC, Herrmann FR, Gold G, Michel JP, Rizzoli R. Does the mini nutritional assessment predict hospitalization outcomes in older people? Age Ageing 2001 May;30(3):221–226. PMID:11443023

18. Shyu Y-IL, Lee H-C. Predictors of nursing home placement and home nursing services utilization by elderly patients after hospital discharge in Taiwan. Journal of advanced nursing England; 2002;38(4):398–406.

19. Therese S. Richmond, Donald R. Kauder, Neville E. Strumpf, Tammy Meredith. Characteristics and outcomes of serious traumatic injury in older adults. 2002; doi: 10.1046/j.1532-5415.2002.50051.x

20. Grossman M., Scaff D.W., Miller D., Reed III J., Hoey B., Anderson III H.L., Morris Jr. J.A., Udekwu P. Functional outcomes in octogenarian trauma. Journal of Trauma - Injury, Infection and Critical Care United States: Lippincott Williams and Wilkins (351 West Camden Street, Baltimore MD 21201-2436, United States); 2003;55(1):26–32. doi: 10.1097/01.TA.0000072109.52351.0D

21. Aharonoff GB, Barsky A, Hiebert R, Zuckerman JD, Koval KJ. Predictors of Discharge to a Skilled Nursing Facility following Hip Fracture Surgery in New York State. Gerontology 2004;50(5):298–302. doi: 10.1159/000079127

22. Bourdel-Marchasson I, Vincent S, Germain C, Salles N, Jenn J, Rasoamanarivo E, Emeriau J-P, Rainfray M, Richard-Harston S. Delirium Symptoms and Low Dietary Intake in Older Inpatients Are Independent Predictors of Institutionalization: A 1-Year Prospective Population-Based Study. The Journals of Gerontology Series A: Biological Sciences and Medical Sciences 2004 Apr 1;59(4):M350–M354. doi: 10.1093/gerona/59.4.M350

23. Cynthia J. Brown, Rebecca J. Friedkin, Sharon K. Inouye. Prevalence and outcomes of low mobility in hospitalized older patients. 2004; doi: 10.1111/j.1532-5415.2004.52354.x

24. Broyles R.W., Khaliq A.A., Robertson M.J. Co-morbidity in an elderly inpatient population: Are the risks of co-existing conditions differentially distributed? Journal of Health and Human Services Administration United States: Southern Public Administration Education Foundation Inc. (2103 Fairway Lane, Harrisburg PA 17112, United States); 2005;28(3–4):398–422.

25. Chang EJ, Edelman LS, Morris SE, Saffle JR. Gender influences on burn outcomes in the elderly. Burns 2005 Feb;31(1):31–35. doi: 10.1016/j.burns.2004.07.011

26. Ganesan K, Pan D, Teklehaimenot S, Norris K. Racial differences in institutionalization after hip fractures: California hospital discharge data. Ethnicity & disease United States; 2005;15(4 Suppl 5):S5-3.

27. Bakhos C, O???Connor J, Kyriakides T, Abou-Nukta F, Bonadies J. Vital Capacity as a Predictor of Outcome in Elderly Patients with Rib Fractures: The Journal of Trauma: Injury, Infection, and Critical Care 2006 Jul;61(1):131–134. doi: 10.1097/01.ta.0000223463.88422.6a

28. Foss N.B., Kristensen M.T., Kehlet H. Prediction of postoperative morbidity, mortality and rehabilitation in hip fracture patients: The cumulated ambulation score. Clinical Rehabilitation United Kingdom: SAGE Publications Ltd (55 City Road, London EC1Y 1SP, United Kingdom); 2006;20(8):701–708. doi: 10.1191/0269215506cre987oa

29. Seematter-Bagnoud L., Wietlisbach V., Yersin B., Bula C.J. Healthcare utilization of elderly persons hospitalized after a noninjurious fall in a Swiss academic medical center. Journal of the American Geriatrics Society United States: Blackwell Publishing Inc. (350 Main Street, Malden MA 02148, United States); 2006;54(6):891–897. doi: 10.1111/j.1532-5415.2006.00743.x

30. Abizanda P., Navarro J.L., Romero L., Leon M., Sanchez-Jurado P.M., Dominguez L. Upper extremity function, an independent predictor of adverse events in hospitalized elderly. Gerontology Switzerland: S. Karger AG (Allschwilerstrasse 10, P.O. Box, Basel CH-4009, Switzerland); 2007;53(5):267–273. doi: 10.1159/000102541

31. Chua M., Hoyle G.E., Soiza R.L. Prognostic implications of hyponatremia in elderly hospitalized patients. Archives of Gerontology and Geriatrics Ireland: Elsevier Ireland Ltd (P.O. Box 85, Limerick, Ireland); 2007;45(3):253–258. doi: 10.1016/j.archger.2006.11.002

32. Kind AJH, Smith MA, Frytak JR, Finch MD. Bouncing back: patterns and predictors of complicated transitions 30 days after hospitalization for acute ischemic stroke. Journal of the American Geriatrics Society Malden, Massachusetts: Wiley-Blackwell; 2007;55(3):365–373. doi: 10.1111/j.1532-5415.2007.01091.x

33. Anpalahan M, Gibson SJ. Geriatric syndromes as predictors of adverse outcomes of hospitalization. Internal medicine journal Australia; 2008;38(1):16–23.

34. Cullum S, Metcalfe C, Todd C, Brayne C. Does depression predict adverse outcomes for older medical inpatients? A prospective cohort study of individuals screened for a trial. Age and Ageing 2008 Oct 1;37(6):690–695. doi: 10.1093/ageing/afn193

35. Fisher AA, Southcott EN, Goh SL, Srikusalanukul W, Hickman PE, Davis MW, Potter JM, Budge MM, Smith PN. Elevated serum cardiac troponin I in older patients with hip fracture: incidence and prognostic significance. Archives of orthopaedic and trauma surgery Germany; 2008;128(10):1073–9. doi: 10.1007/s00402-007-0554-x

36. Marengoni A, Aguero-Torres H, Timpini A, Cossi S, Fratiglioni L. Rehabilitation and nursing home admission after hospitalization in acute geriatric patients. Journal of the American Medical Directors Association United States; 2008;9(4):265–70. doi: 10.1016/j.jamda.2008.01.005

37. Morse B.C., Cobb IV W.S., Valentine J.D., Cass A.L., Roettger R.H. Emergent and elective colon surgery in the extreme elderly: Do the results warrant the operation? American Surgeon United States: Southeastern Surgical Congress (141 West Wieuca Road, Suite B100, Atlanta GA 30342, United States); 2008;74(7):614–618.

38. Bentler SE, Liu L, Obrizan M, Cook EA, Wright KB, Geweke JF, Chrischilles EA, Pavlik CE, Wallace RB, Ohsfeldt RL, Jones MP, Rosenthal GE, Wolinsky FD. The aftermath of hip fracture: discharge placement, functional status change, and mortality. American journal of epidemiology United States; 2009;170(10):1290–9. doi: 10.1093/aje/kwp266

39. Bowles KH, Holmes JH, Ratcliffe SJ, Liberatore M, Nydick R, Naylor MD. Factors Identified by Experts to Support Decision Making for Post Acute Referral. Nursing Research 2009 Mar;58(2):115–122. doi: 10.1097/NNR.0b013e318199b52a

40. Dasgupta M, Rolfson DB, Stolee P, Borrie MJ, Speechley M. Frailty is associated with postoperative complications in older adults with medical problems. Archives of Gerontology and Geriatrics 2009 Jan;48(1):78–83. doi: 10.1016/j.archger.2007.10.007

41. Zekry D, Herrmann FR, Grandjean R, Vitale A-M, De Pinho M-F, Michel J-P, Gold G, Krause K-H. Does dementia predict adverse hospitalization outcomes? A prospective study in aged inpatients. International Journal of Geriatric Psychiatry John Wiley & Sons; 2009;24(3):283–291. doi: 10.1002/gps.2104

42. Aitken LM, Burmeister E, Lang J, Chaboyer W, Richmond TS. Characteristics and Outcomes of Injured Older Adults After Hospital Admission. Journal of the American Geriatrics Society 2010;58(3):442–449. doi: 10.1111/j.1532-5415.2010.02728.x

43. Grover M, Busby-Whitehead J, Palmer MH, Heymen S, Palsson OS, Goode PS, Turner M, Whitehead WE. Survey of geriatricians on the effect of fecal incontinence on nursing home referral. Journal of the American Geriatrics Society Malden, Massachusetts: Wiley-Blackwell; 2010;58(6):1058–1062. doi: 10.1111/j.1532-5415.2010.02863.x

44. Isaia G, Bo M, Aimonino N, Isaia GC, Michelis G, Miceli C, Balcet L, Tizzani A, Zanocchi M. Functional decline two weeks before hospitalization in an elderly population. Aging clinical and experimental research Germany; 2010;22(4):352–5.

45. Kramer AA, Zimmerman JE. Institutional variations in frequency of discharge of elderly intensive care survivors to postacute care facilities. Critical care medicine United States; 2010;38(12):2319–28. doi: 10.1097/CCM.0b013e3181fa02e4

46. Makary MA, Segev DL, Pronovost PJ, Syin D, Bandeen-Roche K, Patel P, Takenaga R, Devgan L, Holzmueller CG, Tian J, Fried LP. Frailty as a Predictor of Surgical Outcomes in Older Patients. Journal of the American College of Surgeons 2010 Jun;210(6):901–908. doi: 10.1016/j.jamcollsurg.2010.01.028

47. Zekry D, Loures Valle BH, Lardi C, Graf C, Michel J-P, Gold G, Krause K-H, Herrmann FR. Geriatrics index of comorbidity was the most accurate predictor of death in geriatric hospital among six comorbidity scores. Journal of clinical epidemiology United States; 2010;63(9):1036–44. doi: 10.1016/j.jclinepi.2009.11.013

48. Kothari A., Phillips S., Bretl T., Block K., Weigel T. Components of geriatric assessments predict thoracic surgery outcomes. Journal of Surgical Research United States: Academic Press Inc. (1250 Sixth Avenue, San Diego, California CA 92101, United States); 2011;166(1):5–13. doi: 10.1016/j.jss.2010.05.050

49. Robinson TN, Wallace JI, Wu DS, Wiktor A, Pointer LF, Pfister SM, Sharp TJ, Buckley MJ, Moss M. Accumulated frailty characteristics predict postoperative discharge institutionalization in the geriatric patient. Journal of the American College of Surgeons United States; 2011;213(1):37–4. doi: 10.1016/j.jamcollsurg.2011.01.056

50. Smyth D, Connor S. Identification of factors associated with a need for rehabilitation for patients over 65 years following admission to a general and vascular surgical service. Australasian journal on ageing Australia; 2011;30(4):191–5. doi: 10.1111/j.1741-6612.2010.00475.x

51. Vochteloo A.J.H., Borger Van Der Burg B.L., Mertens B.J.A., Niggebrugge A.H.P., De Vries M.R., Tuinebreijer W.E., Bloem R.M., Nelissen R.G.H.H., Pilot P. Outcome in hip fracture patients related to anemia at admission and allogeneic blood transfusion: An analysis of 1262 surgically treated patients. BMC Musculoskeletal Disorders United Kingdom: BioMed Central Ltd. (Floor 6, 236 Gray's Inn Road, London WC1X 8HB, United Kingdom); 2011;12((Vochteloo, Bloem, Pilot) Department

of Orthopaedics, Reinier de Graaf Group, PO Box 5011, 2600 GA Delft, Netherlands(Vochteloo, Nelissen) Department of Orthopaedics, Leiden University, Medical Center, PO Box 9600, 2300 RC Leiden, Netherlands(Borger Van D):262. doi: 10.1186/1471-2474-12-262

52. Cohen R-R, Lagoo-Deenadayalan SA, Heflin MT, Sloane R, Eisen I, Thacker JM, Whitson HE. Exploring predictors of complication in older surgical patients: a deficit accumulation index and the Braden Scale. Journal of the American Geriatrics Society United States; 2012;60(9):1609–15. doi: 10.1111/j.1532-5415.2012.04109.x

53. Gambier N, Simoneau G, Bihry N, Delcey V, Champion K, Sellier P, Jarrin I, Bergmann J-F, Mouly S. Efficacy of early clinical evaluation in predicting direct home discharge of elderly patients after hospitalization in internal medicine. Southern medical journal United States; 2012;105(2):63–7. doi: 10.1097/SMJ.0b013e318242d74d

54. Henry L, Halpin L, Hunt S, Holmes SD, Ad N. Patient disposition and long-term outcomes after valve surgery in octogenarians. The Annals of thoracic surgery Netherlands; 2012;94(3):744–50. doi: 10.1016/j.athoracsur.2012.04.073

55. Kristensen MT, Kehlet H. Most patients regain prefracture basic mobility after hip fracture surgery in a fast-track programme. Danish medical journal Denmark; 2012;59(6):A4447.

56. Nath SB, Marcus SC. Impact of psychotic disorders on discharge dispositions of adults 65 or older after a general medical inpatient stay. Psychiatr Serv 2012 Apr;63(4):333–337. PMID:22337008

57. Ostir GV, Berges I, Kuo Y-F, Goodwin JS, Ottenbacher KJ, Guralnik JM. Assessing gait speed in acutely ill older patients admitted to an acute care for elders hospital unit. Archives of internal medicine United States; 2012;172(4):353–8. doi: 10.1001/archinternmed.2011.1615

58. Vochteloo AJH, van Vliet-Koppert ST, Maier AB, Tuinebreijer WE, Röling ML, de Vries MR, Bloem RM, Nelissen RGHH, Pilot P. Risk factors for failure to return to the pre-fracture place of residence after hip fracture: a prospective longitudinal study of 444 patients. Arch Orthop Trauma Surg 2012 Jun 1;132(6):823–830. doi: 10.1007/s00402-012-1469-8

59. Brian D. Badgwell, Jordan Stanley, George J. Chang, Matthew Harold Katz, Heather Lin, Jing Ning, Suzanne Klimberg, Janice Nicole Cormier. Comprehensive geriatric assessment of risk factors associated with adverse outcomes and resource utilization in cancer patients undergoing abdominal surgery. 2013; doi: 10.1002/jso.23369

60. Jones TS, Dunn CL, Wu DS, Cleveland JC Jr, Kile D, Robinson TN. Relationship Between Asking an Older Adult About Falls and Surgical Outcomes. JAMA Surgery 2013 Dec 1;148(12):1132–1138. doi: 10.1001/jamasurg.2013.2741

61. Lakhan P, Jones M, Wilson A, Gray LC. The Higher Care At Discharge Index (HCDI): identifying older patients at risk of requiring a higher level of care at discharge. Archives of gerontology and geriatrics Netherlands; 2013;57(2):184–91. doi: 10.1016/j.archger.2013.04.003

62. Neufeld K.J., Leoutsakos J.-M.S., Sieber F.E., Wanamaker B.L., Gibson Chambers J.J., Rao V., Schretlen D.J., Needham D.M. Outcomes of early delirium diagnosis after general anesthesia in the elderly. Anesthesia and Analgesia United States: Lippincott Williams and Wilkins (530 Walnut Street,P O Box 327, Philadelphia PA 19106-3621, United States); 2013;117(2):471–478. doi: 10.1213/ANE.0b013e3182973650

63. Sheean P.M., Peterson S.J., Chen Y., Liu D., Lateef O., Braunschweig C.A. Utilizing multiple methods to classify malnutrition among elderly patients admitted to the medical and surgical intensive care units (ICU). Clinical Nutrition United Kingdom: Churchill Livingstone (1-3 Baxter's Place, Leith Walk, Edinburgh EH1 3AF, United Kingdom); 2013;32(5):752–757. doi: 10.1016/j.clnu.2012.12.012

64. Vochteloo A.J.H., Borger van der Burg B.L., Tuinebreijer W.E., de Vries M.R., Niggebrugge A.H., Bloem R.M., Maier A.B., Nelissen R.G., Pilot P. Do clinical characteristics and outcome in nonagenarians with a hip fracture differ from younger patients? Geriatrics and Gerontology International Australia: Blackwell Publishing (550 Swanston Street, Carlton South VIC 3053, Australia); 2013;13(1):190–197. doi: 10.1111/j.1447-0594.2012.00885.x

65. Costa AP, Hirdes JP, Heckman GA, Dey AB, Jonsson PV, Lakhan P, Ljunggren G, Singler K, Sjostrand F, Swoboda W, Wellens NIH, Gray LC. Geriatric Syndromes Predict Postdischarge Outcomes Among Older Emergency Department Patients: Findings From the interRAI Multinational Emergency Department Study. Pines J, editor. Acad Emerg Med 2014 Apr;21(4):422–433. doi: 10.1111/acem.12353

66. De Buyser SL, Petrovic M, Taes YE, Vetrano DL, Onder G. A multicomponent approach to identify predictors of hospital outcomes in older in-patients: a multicentre, observational study. PloS one United States; 2014;9(12):e115413. doi: 10.1371/journal.pone.0115413

67. Dent E, Hoogendijk EO. Psychosocial factors modify the association of frailty with adverse outcomes: a prospective study of hospitalised older people. BMC geriatrics England; 2014;14(100968548):108. doi: 10.1186/1471-2318-14-108

68. Hashimoto M, Matsuzaki Y, Kawahara K, Matsuda H, Nishimura G, Hatae T, Kimura Y, Arai K. Medication-related factors affecting discharge to home. Biological & pharmaceutical bulletin Japan; 2014;37(7):1228–33.

69. Joseph B, Pandit V, Zangbar B, Kulvatunyou N, Hashmi A, Green DJ, O'Keeffe T, Tang A, Vercruysse G, Fain MJ, Friese RS, Rhee P. Superiority of Frailty Over Age in Predicting Outcomes Among Geriatric Trauma Patients: A Prospective Analysis. JAMA Surgery 2014 Aug 1;149(8):766–772. doi: 10.1001/jamasurg.2014.296

70. Kim SW, Han HS, Jung HW, Kim KI, Hwang DW, Kang SB. Multidimensional frailty score for the prediction of postoperative mortality risk. JAMA Surgery 2014;149. doi: 10.1001/jamasurg.2014.241

71. Kuys SS, Crouch T, Dolecka UE, Steele M, Low Choy NL. Use and validation of the Balance Outcome Measure for Elder Rehabilitation in acute care. New Zealand Journal of Physiotherapy New Zealand Society of Physiotherapists; 2014;42(1):16–21.

72. Romero-Ortuno R, O'Dwyer C, Byrne D, O'Riordan D, Silke B. A Risk Index for Geriatric Acute Medical Admissions (RIGAMA). Acute medicine England; 2014;13(1):6–11.

73. Rose M, Pan H, Levinson MR, Staples M. Can frailty predict complicated care needs and length of stay?. Internal medicine journal Australia; 2014;44(8):800–5. doi: 10.1111/imj.12502

74. Ambler GK, Brooks DE, Al Zuhir N, Ali A, Gohel MS, Hayes PD. Effect of frailty on short- and mid-term outcomes in vascular surgical patients. Br J Surg 2015;102. doi: 10.1002/bjs.9785

75. de Guise E, LeBlanc J, Dagher J, Tinawi S, Lamoureux J, Marcoux J, Maleki M, Feyz M. Traumatic brain injury in the elderly: A level 1 trauma centre study. Brain Injury Philadelphia, Pennsylvania: Taylor & Francis Ltd; 2015;29(5):558–564. doi: 10.3109/02699052.2014.976593

76. Korc-Grodzicki B, Sun SW, Zhou Q, Iasonos A, Lu B, Root JC, Downey RJ, Tew WP. Geriatric Assessment as a Predictor of Delirium and Other Outcomes in Elderly Patients With Cancer. Annals of surgery United States; 2015;261(6):1085–90. doi: 10.1097/SLA.0000000000000742

77. Lees MC, Merani S, Tauh K, Khadaroo RG. Perioperative factors predicting poor outcome in elderly patients following emergency general surgery: a multivariate regression analysis. Canadian journal of surgery Journal canadien de chirurgie Canada; 2015;58(5):312–7.

78. Sacks GD, Lawson EH, Dawes AJ, Gibbons MM, Zingmond DS, Ko CY. Which Patients Require More Care after Hospital Discharge? An Analysis of Post-Acute Care Use among Elderly Patients Undergoing Elective Surgery. J Am Coll Surg 2015 Jun;220(6):1113-1121.e2. PMID:25872686

79. Alexander K, Shahrokni A, Mahmoudzadeh Pournaki S, Korc-Grodzicki B. Skilled care utilization after abdominal and pelvic cancer surgery in older patients. European Geriatric Medicine 2016 Sep 1;7(5):438–442. doi: 10.1016/j.eurger.2016.06.007

80. Baek SH, Lee SW, Kim S-W, Ahn SY, Yu M-Y, Kim K-I, Chin HJ, Na KY, Chae D-W, Kim S. Frailty as a Predictor of Acute Kidney Injury in Hospitalized Elderly Patients: A Single Center, Retrospective Cohort Study. PloS one United States; 2016;11(6):e0156444. doi: 10.1371/journal.pone.0156444

81. Bellelli G, Mazzone A, Morandi A, Latronico N, Perego S, Zazzetta S, Mazzola P, Annoni G. The Effect of an Impaired Arousal on Short- and Long-Term Mortality of Elderly Patients Admitted to an Acute Geriatric Unit. Journal of the American Medical Directors Association 2016 Mar;17(3):214–219. doi: 10.1016/j.jamda.2015.10.002

82. Chauhan D, Haik N, Merlo A, Haik BJ, Chen C, Cohen M, Mosenthal A, Russo M. Quantitative increase in frailty is associated with diminished survival after transcatheter aortic valve replacement. American Heart Journal 2016 Dec;182:146–154. doi: 10.1016/j.ahj.2016.06.028

83. Cooper Z, Rogers SO, Ngo L, Guess J, Schmitt E, Jones RN, Ayres DK, Walston JD, Gill TM, Gleason LJ, Inouye SK, Marcantonio ER. Comparison of Frailty Measures as Predictors of Outcomes After Orthopedic Surgery. J Am Geriatr Soc 2016 Dec;64(12):2464–2471. doi: 10.1111/jgs.14387

84. Drazin D., Al-Khouja L., Lagman C., Ugiliweneza B., Shweikeh F., Johnson J.P., Kim T.T., Boakye M. Scoliosis surgery in the elderly: Complications, readmissions, reoperations and mortality. Journal of Clinical Neuroscience United Kingdom: Churchill Livingstone; 2016;34((Drazin, Al-Khouja, Lagman, Shweikeh, Johnson) Department of Neurosurgery, Cedars-Sinai Medical Center, 127 S. San Vicente Blvd, Los Angeles, CA 90048, United States(Kim) Department of Orthopedics, Cedars-Sinai Medical Center, Los Angeles, CA, United Stat):158–161. doi: 10.1016/j.jocn.2016.06.005

85. Gibson C.D., Yudelevich E., Jean R.A., Ochieng P., Jean R.E. Assessment of mortality risk in elderly persons with obstructive sleep apnea diagnosed with pneumonia. Current Respiratory Medicine Reviews Netherlands: Bentham Science Publishers B.V. (P.O. Box 294, Bussum 1400 AG, Netherlands); 2016;12(4):299–305. doi: 10.2174/1573398X12666161025111557

86. Hodgman EI, Joseph B, Mohler J, Wolf SE, Paulk ME, Rhodes RL, Nakonezny PA, Phelan HA. Creation of a decision aid for goal setting after geriatric burns: a study from the prognostic assessment of life and limitations after trauma in the elderly [PALLIATE] consortium. The journal of trauma and acute care surgery United States; 2016;81(1):168–72. doi: 10.1097/TA.0000000000000998

87. Kim S., Marsh A.P., Rustowicz L., Roach C., Leng X.I., Kritchevsky S.B., Jack Rejeski W., Groban L. Self-reported mobility in older patients predicts early postoperative outcomes after elective noncardiac surgery. Anesthesiology United States: Lippincott Williams and Wilkins (E-mail: kathiest.clai@apta.org); 2016;124(4):815–825. doi: 10.1097/ALN.0000000000001011

88. Liu SK, Montgomery J, Yan Y, Mecchella JN, Bartels SJ, Masutani R, Batsis JA. Association Between Hospital Admission Risk Profile Score and Skilled Nursing or Acute Rehabilitation Facility Discharges in Hospitalized Older Adults. J Am Geriatr Soc 2016 Oct;64(10):2095–2100. doi: 10.1111/jgs.14345

89. McRae PJ, Walker PJ, Peel NM, Hobson D, Parsonson F, Donovan P, Reade MC, Marquart L, Mudge AM. Frailty and Geriatric Syndromes in Vascular Surgical Ward Patients. Annals of vascular surgery Netherlands; 2016;35(avs, 8703941):9–18. doi: 10.1016/j.avsg.2016.01.033

90. Adogwa O., Elsamadicy A.A., Fialkoff J., Cheng J., Karikari I.O., Bagley C. Early Ambulation Decreases Length of Hospital Stay, Perioperative Complications and Improves Functional Outcomes in Elderly Patients Undergoing Surgery for Correction of Adult Degenerative Scoliosis. Spine United States: Lippincott Williams and Wilkins (E-mail: kathiest.clai@apta.org); 2017;42(18):1420–1425. doi: 10.1097/BRS.0000000000002189

91. Bekelis K, Gottlieb DJ, Su Y, O'Malley AJ, Labropoulos N, Goodney P, Lawton MT, MacKenzie TA. Comparison of clipping and coiling in elderly patients with unruptured cerebral aneurysms. JNS 2017 Mar;126(3):811–818. doi: 10.3171/2016.1.JNS152028

92. Cheung S.C., Ahmad L.A., Hardy J.E., Hilmer S.N. A prospective cohort study of older surgical inpatients examining the prevalence and implications of frailty. Journal of Clinical Gerontology and Geriatrics Netherlands: Content Ed Net Taiwan Limited (E-mail: cathy.kao@contentednet.com); 2017;8(2):71–76. doi: 10.24816/jcgg.2017.v8i2.06

93. Gleason LJ, Benton EA, Alvarez-Nebreda ML, Weaver MJ, Harris MB, Javedan H. FRAIL Questionnaire Screening Tool and Short-Term Outcomes in Geriatric Fracture Patients. Journal of the American Medical Directors Association 2017 Dec;18(12):1082–1086. doi: 10.1016/j.jamda.2017.07.005

94. Hubbard RE, Peel NM, Samanta M, Gray LC, Mitnitski A, Rockwood K. Frailty status at admission to hospital predicts multiple adverse outcomes. Age and ageing England; 2017;46(5):801–806. doi: 10.1093/ageing/afx081

95. Jawa R.S., Singer A.J., Rutigliano D.N., McCormack J.E., Huang E.C., Shapiro M.J., Fields S.D., Morelli B.N., Vosswinkel J.A. Spinal Fractures in Older Adult Patients Admitted After Low-Level Falls: 10-Year Incidence and Outcomes. Journal of the American Geriatrics Society United States: Blackwell Publishing Inc. (E-mail: subscrip@blackwellpub.com); 2017;65(5):909–915. doi: 10.1111/jgs.14669

96. Joseph B., Orouji Jokar T., Hassan A., Azim A., Mohler M.J., Kulvatunyou N., Siddiqi S., Phelan H., Fain M., Rhee P. Redefining the association between old age and poor outcomes after trauma: The impact of frailty syndrome. Journal of Trauma and Acute Care Surgery United States: Lippincott Williams and Wilkins (E-mail: kathiest.clai@apta.org); 2017;82(3):575–581. doi: 10.1097/TA.0000000000001329

97. Khan M, O'Keeffe T, Jehan F, Kulvatunyou N, Kattaa A, Gries L, Tang A, Joseph B. The impact of Glasgow Coma Scale-age prognosis score on geriatric traumatic brain injury outcomes. The Journal of surgical research United States; 2017;216(k7b, 0376340):109–114. doi: 10.1016/j.jss.2017.04.026

98. McIsaac DI, Moloo H, Bryson GL, van Walraven C. The Association of Frailty With Outcomes and Resource Use After Emergency General Surgery: A Population-Based Cohort Study. Anesthesia & Analgesia 2017 May;124(5):1653–1661. doi: 10.1213/ANE.0000000000001960

99. Mitchell R., Harvey L., Draper B., Brodaty H., Close J. Risk factors associated with residential aged care, respite and transitional aged care admission for older people following an injury-related hospitalisation. Archives of Gerontology and Geriatrics Ireland: Elsevier Ireland Ltd; 2017;72((Mitchell) Australian Institute of Health Innovation, Macquarie University, Australia(Harvey, Close) Falls, Balance and Injury Research Centre, Neuroscience Research Australia, University of New South Wales, Australia(Draper, Brodaty) Dementia Collaborati):59–66. doi: 10.1016/j.archger.2017.05.012

100. Pelavski AD, De Miguel M, Alcaraz Garcia-Tejedor G, Villarino L, Lacasta A, Señas L, Rochera MI. Mortality, Geriatric, and Nongeriatric Surgical Risk Factors Among the Eldest Old: A Prospective Observational Study. Anesthesia & Analgesia 2017 Oct;125(4):1329–1336. doi: 10.1213/ANE.0000000000002389

101. Rajaee S.S., Yalamanchili D., Noori N., Debbi E., Mirocha J., Lin C.A., Moon C.N. Increasing use of reverse total shoulder arthroplasty for proximal humerus fractures in elderly patients. Orthopedics United States: Slack Incorporated (E-mail: customerservice@slackinc.com); 2017;40(6):e982–e989. doi: 10.3928/01477447-20170925-01

102. Romero-Ortuno R., Forsyth D.R., Wilson K.J., Cameron E., Wallis S., Biram R., Keevil V. The association of geriatric syndromes with hospital outcomes. Journal of Hospital Medicine United States: Society of hospital medicine (E-mail: jhospitalmedicine@jjeditorial.com); 2017;12(2):83–89. doi: 10.12788/jhm.2685

103. Adogwa O, Elsamadicy AA, Sergesketter A, Vuong VD, Moreno J, Cheng J, Karikari IO, Bagley CA. Independent Association Between Preoperative Cognitive Status and Discharge Location After Surgery: A Strategy to Reduce Resource Use After Surgery for Deformity. World Neurosurgery 2018 Feb;110:e67–e72. doi: 10.1016/j.wneu.2017.10.081

104. Arshi A, Lai WC, Chen JB, Bukata SV, Stavrakis AI, Zeegen EN. Predictors and Sequelae of Postoperative Delirium in Geriatric Hip Fracture Patients. Geriatr Orthop Surg Rehabil 2018 Jan 1;9:215145931881482. doi: 10.1177/2151459318814823

105. Black S, Nicholas C, Cotton S, Brock K. Determining discharge destination in geriatric evaluation and management units: Is progressive goal attainment a better early indicator of discharge destination than improvement in functional independence measure scores?: Determining discharge destination in GEM. Geriatr Gerontol Int 2018 Jul;18(7):1058–1063. doi: 10.1111/ggi.13308

106. Choi K.S., Jeong Y.M., Lee E., Kim K.I., Yee J., Lee B.K., Chung J.E., Rhie S.J., Gwak H.S. Association of pre-operative medication use with post-surgery mortality and morbidity in oncology patients receiving comprehensive geriatric assessment. Aging Clinical and Experimental Research Switzerland: Springer International Publishing; 2018;30(10):1177–1185. doi: 10.1007/s40520-018-0904-2

107. Curtis E, Romanowski K, Sen S, Hill A, Cocanour C. Frailty score on admission predicts mortality and discharge disposition in elderly trauma patients over the age of 65 y. The Journal of surgical research United States; 2018;230(k7b, 0376340):13–19. doi: 10.1016/j.jss.2018.04.017

108. Garcia-Ptacek S, Contreras Escamez B, Zupanic E, Religa D, von Koch L, Johnell K, von Euler M, Kareholt I, Eriksdotter M. Prestroke Mobility and Dementia as Predictors of Stroke Outcomes in Patients Over 65 Years of Age: A Cohort Study From The Swedish Dementia and Stroke Registries. Journal of the American Medical Directors Association United States; 2018;19(2):154–161. doi: 10.1016/j.jamda.2017.08.014

109. HALEVI AE, MAUER E, SALDINGER P, HAGLER DJ. Predictors of Dependency in Geriatric Trauma Patients with Rib Fractures: A Population Study. American Surgeon Westwood, Kansas: Southeastern Surgical Congress; 2018;84(12):1856–1860. doi: 10.1177/000313481808401228

110. Iida H., Sakai Y., Watanabe T., Matsui H., Takemura M., Matsui Y., Harada A., Hida T., Ito K., Ito S. Sarcopenia affects conservative treatment of osteoporotic vertebral fracture. Osteoporosis and Sarcopenia South Korea: Korean Society of Osteoporosis; 2018;4(3):114–117. doi: 10.1016/j.afos.2018.09.002

111. Karlsson E, Egenvall M, Farahnak P, Bergenmar M, Nygren-Bonnier M, Franzen E, Rydwik E. Better preoperative physical performance reduces the odds of complication severity and discharge to care facility after abdominal cancer resection in people over the age of 70 - A prospective cohort study. European journal of

surgical oncology : the journal of the European Society of Surgical Oncology and the British Association of Surgical Oncology England; 2018;44(11):1760–1767. doi: 10.1016/j.ejso.2018.08.011

112. Lucke JA, van der Mast RC, de Gelder J, Heim N, de Groot B, Mooijaart SP, Blauw GJ. The Six-Item Cognitive Impairment Test Is Associated with Adverse Outcomes in Acutely Hospitalized Older Patients: A Prospective Cohort Study. Dementia and geriatric cognitive disorders extra Switzerland; 2018;8(2):259–267. doi: 10.1159/000490240

113. Racine AM, Fong TG, Gou Y, Travison TG, Tommet D, Erickson K, Jones RN, Dickerson BC, Metzger E, Marcantonio ER, Schmitt EM, Inouye SK. Clinical outcomes in older surgical patients with mild cognitive impairment. Alzheimer’s & Dementia 2018 May;14(5):590–600. doi: 10.1016/j.jalz.2017.10.010

114. Sinvani L, Kozikowski A, Patel V, Mulvany C, Smilios C, Qiu G, Zhang M, Wolf-Klein G, Pekmezaris R. Measuring Functional Status in Hospitalized Older Adults Through Electronic Health Record Documentation. Southern medical journal United States; 2018;111(4):220–225. doi: 10.14423/SMJ.0000000000000788

115. Wang HT, Fafard J, Ahern S, Vendittoli P-A, Hebert P. Frailty as a predictor of hospital length of stay after elective total joint replacements in elderly patients. BMC Musculoskelet Disord 2018 Dec;19(1):14. doi: 10.1186/s12891-018-1935-8

116. Amabili P, Wozolek A, Noirot I, Roediger L, Senard M, Donneau A-F, Hubert MB, Brichant J-F, Hans GA. The Edmonton Frail Scale Improves the Prediction of 30-Day Mortality in Elderly Patients Undergoing Cardiac Surgery: A Prospective Observational Study. Journal of Cardiothoracic and Vascular Anesthesia 2019 Apr 1;33(4):945–952. doi: 10.1053/j.jvca.2018.05.038

117. Barry RG, Wolbert TT, Mozaffari FB, Ray PD, Thompson EC, Gress TW, Denning DA. Comparison of Geriatric Trauma Outcomes When Admitted to a Medical or Surgical Service After a Fall. The Journal of surgical research United States; 2019;233(k7b, 0376340):391–396. doi: 10.1016/j.jss.2018.08.037

118. Birkelbach O., Morgeli R., Spies C., Olbert M., Weiss B., Brauner M., Neuner B., Francis R.C.E., Treskatsch S., Balzer F. Routine frailty assessment predicts postoperative complications in elderly patients across surgical disciplines - A retrospective observational study. BMC Anesthesiology United Kingdom: BioMed Central Ltd. (E-mail: info@biomedcentral.com); 2019;19(1):204. doi: 10.1186/s12871-019-0880-x

119. de Jong L, van Rijckevorsel VAJIM, Raats JW, Klem TMAL, Kuijper TM, Roukema GR. Delirium after hip hemiarthroplasty for proximal femoral fractures in elderly patients: risk factors and clinical outcomes. Clinical interventions in aging New Zealand; 2019;14(101273480):427–435. doi: 10.2147/CIA.S189760

120. Hamidi M., Zeeshan M., Leon-Risemberg V., Nikolich-Zugich J., Hanna K., Kulvatunyou N., Saljuqi A.T., Fain M., Joseph B. Frailty as a prognostic factor for the critically ill older adult trauma patients. American Journal of Surgery United States: Elsevier Inc. (E-mail: usjcs@elsevier.com); 2019;218(3):484–489. doi: 10.1016/j.amjsurg.2019.01.035

121. Hamidi M, Haddadin Z, Zeeshan M, Saljuqi AT, Hanna K, Tang A, Northcutt A, Kulvatunyou N, Gries L, Joseph B. Prospective evaluation and comparison of the predictive ability of different frailty scores to predict outcomes in geriatric trauma patients. The journal of trauma and acute care surgery United States; 2019;87(5):1172–1180. doi: 10.1097/TA.0000000000002458

122. Hulsbæk S, Larsen RF, Rosthøj S, Kristensen MT. The Barthel Index and the Cumulated Ambulation Score are superior to the de Morton Mobility Index for the early assessment of outcome in patients with a hip fracture admitted to an acute geriatric ward. Disability and Rehabilitation 2019 May 22;41(11):1351–1359. doi: 10.1080/09638288.2018.1424951

123. Inoue T, Misu S, Tanaka T, Kakehi T, Kakiuchi M, Chuman Y, Ono R. Frailty defined by 19 items as a predictor of short-term functional recovery in patients with hip fracture. Injury Netherlands; 2019;50(12):2272–2276. doi: 10.1016/j.injury.2019.10.011

124. Janssen T.L., Hosseinzoi E., Vos D.I., Veen E.J., Mulder P.G.H., Van Der Holst A.M., Van Der Laan L. The importance of increased awareness for delirium in elderly patients with rib fractures after blunt chest wall trauma: A retrospective cohort study on risk factors and outcomes. BMC Emergency Medicine United Kingdom: BioMed Central Ltd. (E-mail: info@biomedcentral.com); 2019;19(1):34. doi: 10.1186/s12873-019-0248-z

125. Malik AT, Quatman CE, Phieffer LS, Ly TV, Wiseman J, Khan SN. The impact of metabolic syndrome on 30-day outcomes in geriatric hip fracture surgeries. European journal of orthopaedic surgery & traumatology : orthopedie traumatologie France; 2019;29(2):427–433. doi: 10.1007/s00590-018-2298-4

126. McIsaac DI, Wong CA, Huang A, Moloo H, van Walraven C. Derivation and Validation of a Generalizable Preoperative Frailty Index Using Population-based Health Administrative Data. Ann Surg 2019 Jul;270(1):102–108. PMID:29672410

127. Meyer AM, Becker I, Siri G, Brinkkötter PT, Benzing T, Pilotto A, Polidori MC. New associations of the Multidimensional Prognostic Index. Z Gerontol Geriat 2019 Aug;52(5):460–467. doi: 10.1007/s00391-018-01471-6

128. Sato M, Ido Y, Yoshimura Y, Mutai H. Relationship of Malnutrition During Hospitalization With Functional Recovery and Postdischarge Destination in Elderly Stroke Patients. Journal of stroke and cerebrovascular diseases : the official journal of National Stroke Association United States; 2019;28(7):1866–1872. doi: 10.1016/j.jstrokecerebrovasdis.2019.04.012

129. Scharf A-C, Gronewold J, Dahlmann C, Schlitzer J, Kribben A, Gerken G, Rassaf T, Kleinschnitz C, Dodel R, Frohnhofen H, Hermann DM. Health outcome of older hospitalized patients in internal medicine environments evaluated by Identification of Seniors at Risk (ISAR) screening and geriatric assessment. BMC geriatrics England; 2019;19(1):221. doi: 10.1186/s12877-019-1239-3

130. Spannella F, Giulietti F, Cocci G, Landi L, Lombardi FE, Borioni E, Cenci A, Giordano P, Sarzani R. Acute Exacerbation of Chronic Obstructive Pulmonary Disease in Oldest Adults: Predictors of In-Hospital Mortality and Need for Post-acute Care. Journal of the American Medical Directors Association United States; 2019;20(7):893–898. doi: 10.1016/j.jamda.2019.01.125

131. Trostrup J, Andersen H, Kam CAM, Magnusson SP, Beyer N. Assessment of Mobility in Older People Hospitalized for Medical Illness Using the de Morton Mobility Index and Cumulated Ambulation Score-Validity and Minimal Clinical Important Difference. Journal of geriatric physical therapy (2001) United States; 2019;42(3):153–160. doi: 10.1519/JPT.0000000000000170

132. Wang J, Dietrich MS, Bell SP, Maxwell CA, Simmons SF, Kripalani S, Vanderbilt Inpatient Cohort Study (VICS). Changes in vulnerability among older patients with cardiovascular disease in the first 90 days after hospital discharge: A secondary analysis of a cohort study. BMJ open England; 2019;9(1):e024766. doi: 10.1136/bmjopen-2018-024766

133. Warren J.A., Sundaram K., Anis H.K., Piuzzi N.S., Higuera C.A., Kamath A.F. Total Hip Arthroplasty Outperforms Hemiarthroplasty in Patients Aged 65 Years and Older: A Propensity-Matched Study of Short-Term Outcomes. Geriatric Orthopaedic Surgery and Rehabilitation United States: SAGE Publications Inc. (E-mail: claims@sagepub.com); 2019;10((Warren, Sundaram, Anis, Piuzzi, Higuera, Kamath) Department of Orthopaedic Surgery, Orthopaedic and Rheumatology Institute, Cleveland Clinic, Cleveland, OH, United States). doi: 10.1177/2151459319876854

134. Wiegand A, Zieger A, Staiger RD, Egli A, Freystätter G, Bischoff-Ferrari HA, Chocano-Bedoya PO. Association of depression with malnutrition, grip strength and impaired cognitive function among senior trauma patients. Journal of Affective Disorders Elsevier B.V.; 2019;243:175–182. doi: 10.1016/j.jad.2019.01.013

135. Abraham A, Burrows S, Abraham NJ, Mandal B. Modified frailty index and hypoalbuminemia as predictors of adverse outcomes in older adults presenting to acute general surgical unit. Revista espanola de geriatria y gerontologia Spain; 2020;55(2):70–75. doi: 10.1016/j.regg.2019.09.005

136. Arshi A, Iglesias BC, Zambrana LE, Lai WC, Zeegen EN, Sassoon AA, Stavrakis AI. Postacute Care Utilization in Postsurgical Orthogeriatric Hip Fracture Care. J Am Acad Orthop Surg 2020 Sep 15;28(18):743–749. doi: 10.5435/JAAOS-D-19-00073

137. Eichler S, Völler H, Reibis R, Wegscheider K, Butter C, Harnath A, Salzwedel A. Geriatric or cardiac rehabilitation? Predictors of treatment pathways in advanced age patients after transcatheter aortic valve implantation. BMC Cardiovasc Disord 2020 Dec;20(1):158. doi: 10.1186/s12872-020-01452-x

138. Gamboa-Arango A, Duaso E, Malafarina V, Formiga F, Marimon P, Sandiumenge M, Salgado M-T, Escalante E, Lumbreras C, Tarrida A. Prognostic factors for discharge to home and residing at home 12 months after hip fracture: an Anoia hip study. Aging clinical and experimental research Germany; 2020;32(5):925–933. doi: 10.1007/s40520-019-01273-9

139. Gazzotti A., Meyer U., Freystaetter G., Palzer M., Theiler R., Abderhalden L., Bischoff-Ferrari H.A. Physical performance among patients aged 70 + in acute care: a preliminar comparison between the Short Physical Performance Battery and the De Morton Mobility Index with regard to sensitivity to change and prediction of discharge destination. Aging Clinical and Experimental Research Switzerland: Springer; 2020;32(4):579–586. doi: 10.1007/s40520-019-01249-9

140. Hagg S., Jylhava J., Wang Y., Xu H., Metzner C., Annetorp M., Garcia-Ptacek S., Khedri M., Bostrom A.-M., Kadir A., Johansson A., Kivipelto M., Eriksdotter M., Cederholm T., Religa D. Age, Frailty, and Comorbidity as Prognostic Factors for Short-Term Outcomes in Patients With Coronavirus Disease 2019 in Geriatric Care. Journal of the American Medical Directors Association United States: Elsevier Inc.; 2020;21(11):1555. doi: 10.1016/j.jamda.2020.08.014

141. Lisk R, Yeong K, Enwere P, Jenkinson J, Robin J, Irvin-Sellers M, Fluck D, Osmani A, Sharmin R, Sharma P, Fry CH, Han TS. Associations of 4AT with mobility, length of stay and mortality in hospital and discharge destination among patients admitted with hip fractures. Age and ageing England; 2020;49(3):411–417. doi: 10.1093/ageing/afz161

142. Martin AN, Hoagland DL, Turrentine FE, Jones RS, Zaydfudim VM. Safety of Major Abdominal Operations in the Elderly: A Study of Geriatric-Specific Determinants of Health. World journal of surgery United States; 2020;44(8):2592–2600. doi: 10.1007/s00268-020-05515-0

143. McIsaac DI, Taljaard M, Bryson GL, Beaule PE, Gagne S, Hamilton G, Hladkowicz E, Huang A, Joanisse JA, Lavallee LT, MacDonald D, Moloo H, Thavorn K, van Walraven C, Yang H, Forster AJ. Frailty as a Predictor of Death or New Disability After Surgery: A Prospective Cohort Study. Annals of surgery United States; 2020;271(2):283–289. doi: 10.1097/SLA.0000000000002967

144. Nishioka S., Wakabayashi H., Maeda K., Shamoto H., Taketani Y., Kayashita J., Momosaki R. Body mass index and recovery of activities of daily living in older patients with femoral fracture: An analysis of a national inpatient database in Japan. Archives of Gerontology and Geriatrics Ireland: Elsevier Ireland Ltd; 2020;87((Nishioka) Department of Clinical Nutrition and Food Service, Nagasaki Rehabilitation Hospital, 4-11 Gin-yamachi, Nagasaki City, Nagasaki 850-0854, Japan(Nishioka, Taketani) Department of Clinical Nutrition and Food Management, Institute of Biomedical Sci):104009. doi: 10.1016/j.archger.2020.104009

145. Scheidt S, Gathen M, Lukas A, Welle K, Kohlhof H, Wirtz DC, Burger C, Kabir K. [Challenges of discharge management in geriatric traumatology : Example of an integrated orthogeriatric service]. Der Unfallchirurg Germany; 2020;123(7):534–540. doi: 10.1007/s00113-020-00812-8

146. Susano MJ, Grasfield RH, Friese M, Rosner B, Crosby G, Bader AM, Kang JD, Smith TR, Lu Y, Groff MW, Chi JH, Grodstein F, Culley DJ. Brief Preoperative Screening for Frailty and Cognitive Impairment Predicts Delirium after Spine Surgery. Anesthesiology United States; 2020;133(6):1184–1191. doi: 10.1097/ALN.0000000000003523

147. Tasheva P., Vollenweider P., Kraege V., Roulet G., Lamy O., Marques-Vidal P., Mean M. Association between Physical Activity Levels in the Hospital Setting and Hospital-Acquired Functional Decline in Elderly Patients. JAMA Network Open United States: American Medical Association; 2020;3(1):e1920185. doi: 10.1001/jamanetworkopen.2019.20185

148. Tracy BM, Adams MA, Schenker ML, Gelbard RB. The 5 and 11 Factor Modified Frailty Indices are Equally Effective at Outcome Prediction Using TQIP. Journal of Surgical Research 2020 Nov;255:456–462. doi: 10.1016/j.jss.2020.05.090

149. Wang HT, Carrier FM, Tremblay A, Joly M-M, Ghali R, Heckman G, Hirdes JP, Hebert P. Outcomes of vulnerable elderly patients undergoing elective major surgery: a prospective cohort study. Canadian journal of anaesthesia = Journal canadien d'anesthesie United States; 2020;67(7):847–856. doi: 10.1007/s12630-020-01646-z

150. Warnier RMJ, van Rossum E, van Kuijk SMJ, Magdelijns F, Schols JMGA, Kempen GIJM. Frailty screening in hospitalised older adults: How does the brief Dutch National Safety Management Program perform compared to a more extensive approach? Journal of Clinical Nursing (John Wiley & Sons, Inc) John Wiley & Sons, Inc.; 2020;29(7/8):1064–1073. doi: 10.1111/jocn.15148

151. Zhang LM, Hornor MA, Robinson T, Rosenthal RA, Ko CY, Russell MM. Evaluation of Postoperative Functional Health Status Decline Among Older Adults. JAMA surgery United States; 2020;155(10):950–958. doi: 10.1001/jamasurg.2020.2853

152. Chaudhry YP, Puvanesarajah V, Oni JK, Sterling RS, Khanuja HS. A Fall Within 3 Months Before Total Joint Arthroplasty is Associated With Adverse Outcomes in Elderly Patients. The Journal of arthroplasty United States; 2021;36(4):1246–1250. doi: 10.1016/j.arth.2020.10.025

153. Doherty WJ, Stubbs TA, Chaplin A, Reed MR, Sayer AA, Witham MD, Sorial AK. Prediction of Postoperative Outcomes Following Hip Fracture Surgery: Independent Validation and Recalibration of the Nottingham Hip Fracture Score. Journal of the American Medical Directors Association United States; 2021;22(3):663-669.e2. doi: 10.1016/j.jamda.2020.07.013

154. Geiss R, Sebaste L, Valter R, Poisson J, Mebarki S, Conti C, Vordos D, Bringuier M, Méjean A, Mongiat-Artus P, Cudennec T, Canoui-Poitrine F, Caillet P, Paillaud E. Complications and Discharge after Radical Cystectomy for Older Patients with Muscle-Invasive Bladder Cancer: The ELCAPA-27 Cohort Study. Cancers MDPI; 2021;13(23):6010. doi: 10.3390/cancers13236010

155. Hung Y-C, Wolf JH, D'Adamo CR, Demos J, Katlic MR, Svoboda S. Preoperative functional status is associated with discharge to nonhome in geriatric individuals. Journal of the American Geriatrics Society United States; 2021;69(7):1856–1864. doi: 10.1111/jgs.17128

156. Iida H., Seki T., Sakai Y., Watanabe T., Wakao N., Matsui H., Imagama S. Low muscle mass affect hip fracture treatment outcomes in older individuals: a single-institution case-control study. BMC Musculoskeletal Disorders United Kingdom: BioMed Central Ltd; 2021;22(1):259. doi: 10.1186/s12891-021-04143-6

157. Kim H.C., An S.B., Jeon H., Kim T.W., Oh J.K., Shin D.A., Yi S., Kim K.N., Lee P.H., Kang S.Y., Ha Y. Preoperative cognitive impairment as a predictor of postoperative outcomes in elderly patients undergoing spinal surgery for degenerative spinal disease. Journal of Clinical Medicine Switzerland: MDPI; 2021;10(7):1385. doi: 10.3390/jcm10071385

158. Luders S., Meis A.-P., Schrader B. Frequency of hypertension and renal insufficiency and their clinical significance for the success of patients in early rehabilitative geriatric complex treatment. Nieren- und Hochdruckkrankheiten Germany: Dustri-Verlag Dr. Karl Feistle; 2021;50(1):32–41. doi: 10.5414/NHX02149

159. McCarty AR, Villarreal ME, Tamer R, Strassels SA, Schubauer KM, Paredes AZ, Santry H, Wisler JR. Analyzing Outcomes Among Older Adults With Necrotizing Soft-Tissue Infections in the United States. The Journal of surgical research United States; 2021;257(k7b, 0376340):107–117. doi: 10.1016/j.jss.2020.06.031

160. Nishijima TF, Esaki T, Morita M, Toh Y. Preoperative frailty assessment with the Robinson Frailty Score, Edmonton Frail Scale, and G8 and adverse postoperative outcomes in older surgical patients with cancer. European journal of surgical oncology : the journal of the European Society of Surgical Oncology and the British Association of Surgical Oncology England; 2021;47(4):896–901. doi: 10.1016/j.ejso.2020.09.031

161. Palzer M, Meyer U, Abderhalden LA, Gazzotti A, Hierholzer C, Bischoff-Ferrari HA, Freystätter G. Geriatrische Komplexbehandlung bei alterstraumatologischen Patienten: Einfluss auf Mobilität, Selbsthilfefähigkeit und Austrittsdestination. Z Gerontol Geriat 2021 Dec;54(8):816–822. doi: 10.1007/s00391-020-01812-4

162. Sanfilippo S., Michaud V., Wei J., Bikmetov R., Turgeon J., Brunetti L. Classification and assessment of medication risk in the elderly (Care): Use of a medication risk score to inform patients' readmission likelihood after hospital discharge. Journal of Clinical Medicine Switzerland: MDPI; 2021;10(17):3947. doi: 10.3390/jcm10173947

163. Schoeneberg C, Pass B, Oberkircher L, Rascher K, Knobe M, Neuerburg C, Lendemans S, Aigner R, Registry for Geriatric Trauma DGU. Impact of concomitant injuries in geriatric patients with proximal femur fracture : an analysis of the Registry for Geriatric Trauma. The bone & joint journal England; 2021;103-B(9):1526–1533. doi: 10.1302/0301-620X.103B9.BJJ-2021-0358.R1

164. Schuijt HJ, Morin ML, Allen E, Weaver MJ. Does the frailty index predict discharge disposition and length of stay at the hospital and rehabilitation facilities?. Injury Netherlands; 2021;52(6):1384–1389. doi: 10.1016/j.injury.2021.01.004

165. Tsai SHL, Lin T-Y, Tischler EH, Hung K-H, Chen C-H, Osgood GM, Fu T-S, Su C-Y. Distal femur fractures have a higher mortality rate compared to hip fractures among the elderly: Insights from the National Trauma Data Bank. Injury Netherlands; 2021;52(7):1903–1907. doi: 10.1016/j.injury.2021.04.023

166. van Dartel D, Vermeer M, Folbert EC, Arends AJ, Vollenbroek-Hutten MMR, Hegeman JH, Dutch Hip Fracture Audit (DHFA) Group. Early Predictors for Discharge to Geriatric Rehabilitation after Hip Fracture Treatment of Older Patients. Journal of the American Medical Directors Association United States; 2021;22(12):2454–2460. doi: 10.1016/j.jamda.2021.03.026

167. Van Der Vliet QMJ, Weaver MJ, Heil K, McTague MF, Heng M. Factors for Increased Hospital Stay and Utilization of Post -Acute Care Facilities in Geriatric Orthopaedic Fracture Patients. The archives of bone and joint surgery Iran; 2021;9(1):70–78. doi: 10.22038/abjs.2020.46476.2276

168. Yeates E.O., Grigorian A., Kuza C.M., Nguyen N.T., Inaba K., Dolich M., Nahmias J. The DEPARTS Score: A Novel Tool for Predicting Discharge Disposition in Geriatric Trauma Patients. American Surgeon United States: SAGE Publications Inc.; 2021;((Yeates, Grigorian, Nguyen, Dolich, Nahmias) Department of Surgery,

University of California Irvine, Orange, CA, United States(Grigorian, Inaba) Department of Surgery, University of Southern California, CA, United States(Kuza) Department of Anesthesiology). doi: 10.1177/00031348211029843

169. Kusen JQ, van der Naald N, van Overeem L, van der Vet PCR, Smeeing DPJ, Eversdijk HAJ, Verleisdonk EJMM, van der Velde D, Schuijt HJ. Is the Parker Mobility Score in the older patient with a traumatic hip fracture associated with discharge disposition after surgery? A retrospective cohort study. Eur J Trauma Emerg Surg 2022 Jun;48(3):1919–1927. PMID:34097075

170. Lai K, Anantha RV, Fawcett V, Tsang B, Kim M, Widder S. Early predictors of discharge to home among severely injured geriatric patients: A single-system retrospective cohort study. Trauma SAGE Publications; 2022 Apr 1;24(2):154–163. doi: 10.1177/1460408620982261

171. Sokas CM, Hu FY, Dalton MK, Jarman MP, Bernacki RE, Bader A, Rosenthal RA, Cooper Z. Understanding the role of informal caregivers in postoperative care transitions for older patients. Journal of the American Geriatrics Society Malden, Massachusetts: Wiley-Blackwell; 2022;70(1):208–217. doi: 10.1111/jgs.17507

172. Tierney AJ, Gloss SJ, Hunter HC, Macmillan MS. Experiences of elderly patients concerning discharge from hospital. J Clin Nurs 1993 May;2(3):179–185. doi: 10.1111/j.1365-2702.1993.tb00158.x

173. Bull MJ. A discharge planning questionnaire for clinical practice. Applied Nursing Research 1994 Nov;7(4):193–199. doi: 10.1016/0897-1897(94)90027-2

174. Bull MJ. Patients' and professionals' perceptions of quality in discharge planning. 1994; doi: 10.1097/00001786-199401000-00009

175. Congdon JG. Managing the incongruities: The hospital discharge experience for elderly patients, their families, and nurses. Applied Nursing Research 1994 Aug;7(3):125–131. doi: 10.1016/0897-1897(94)90004-3

176. Mcwilliam CL, Sangster JF. Managing Patient Discharge to Home: The Challenges of Achieving Quality of Care. International Journal for Quality in Health Care 1994 Jun 1;6(2):147–161. doi: 10.1093/intqhc/6.2.147

177. Bull MJ, Kane RL. Gaps in Discharge Planning. J Appl Gerontol 1996 Dec;15(4):486–500. doi: 10.1177/073346489601500406

178. Eric A. Coleman, Jodi D. Smith, Janet C. Frank, Theresa B. Eilertsen, Jill N. Thiare, Andrew M. Kramer. Development and testing of a measure designed to assess the quality of care transitions. 2002; doi: 10.5334/ijic.60

179. Burke RE, Lawrence E, Ladebue A, Ayele R, Lippmann B, Cumbler E, Allyn R, Jones J. How Hospital Clinicians Select Patients for Skilled Nursing Facilities. J Am Geriatr Soc 2017 Nov;65(11):2466–2472. doi: 10.1111/jgs.14954

180. Isbel ST, Jamieson MI. Views from health professionals on accessing rehabilitation for people with dementia following a hip fracture. Dementia: The International Journal of Social Research and Practice Sage Publications; 2017;16(8):1020–1031. doi: 10.1177/1471301216631141

181. Bradley G, Baker K, Bailey C. Exploring how occupational therapists and physiotherapists evaluate rehabilitation potential of older people in acute care. British Journal of Occupational Therapy SAGE Publications Ltd STM; 2022 Mar 1;85(3):199–207. doi: 10.1177/03080226211011386
